# Zero-Field SMM Behavior Triggered by Magnetic Exchange Interactions and a Collinear Arrangement of Local Anisotropy Axes in a Linear Co_3_^II^ Complex

**DOI:** 10.1021/acs.inorgchem.3c02817

**Published:** 2023-11-22

**Authors:** Andoni Zabala-Lekuona, Aritz Landart-Gereka, María Mar Quesada-Moreno, Antonio J. Mota, Ismael F. Díaz-Ortega, Hiroyuki Nojiri, Jurek Krzystek, José M. Seco, Enrique Colacio

**Affiliations:** † Departamento de Química Aplicada, Facultad de Química, Universidad del País Vasco (UPV/EHU), 20018 Donostia-San Sebastián, Spain; ‡ Departamento de Química Inorgánica, Facultad de Ciencias, Universidad de Granada, 18071 Granada, Spain; § Institute for Materials Research, Tohoku University, Katahira, Sendai 980-8577, Japan; ∥ National High Magnetic Field Laboratory, Florida State University, Tallahassee, Florida 32310, United States

## Abstract

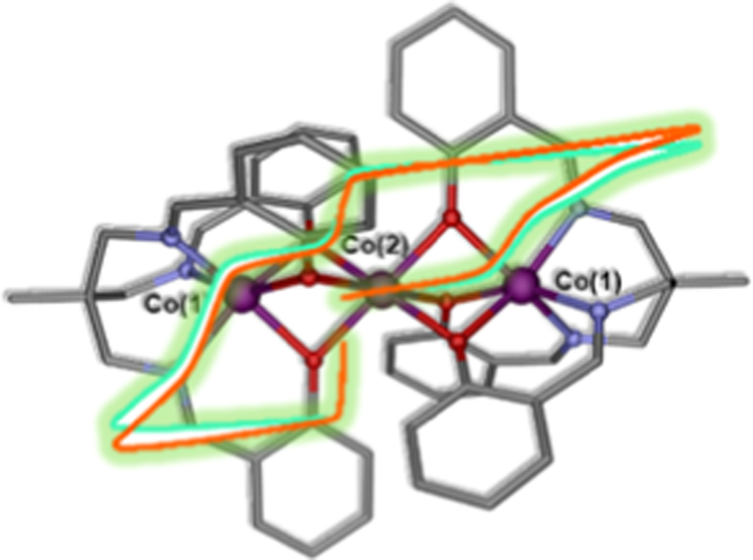

A new linear trinuclear Co(II)_3_ complex with a formula of [{Co(μ-L)}_2_Co] has been prepared by self-assembly of Co(II) ions and the N_3_O_3_-tripodal Schiff base ligand H_3_L, which is obtained from the condensation of 1,1,1-tris(aminomethyl)ethane and salicylaldehyde. Single X-ray diffraction shows that this compound is centrosymmetric with triple-phenolate bridging groups connecting neighboring Co(II) ions, leading to a paddle-wheel-like structure with a pseudo-*C*_*3*_ axis lying in the Co–Co–Co direction. The Co(II) ions at both ends of the Co(II)_3_ molecule exhibit distorted trigonal prismatic CoN_3_O_3_ geometry, whereas the Co(II) at the middle presents an elongated trigonal antiprismatic CoO_6_ geometry. The combined analysis of the magnetic data and theoretical calculations reveal strong easy-axis magnetic anisotropy for both types of Co(II) ions (|*D*| values higher than 115 cm^–1^) with the local anisotropic axes lying on the pseudo-*C*_*3*_ axis of the molecule. The magnetic exchange interaction between the middle and ends Co(II) ions, extracted by using either a Hamiltonian accounting for the isotropic magnetic coupling and ZFS or the Lines’ model, was found to be medium to strong and antiferromagnetic in nature, whereas the interaction between the external Co(II) ions is weak antiferromagnetic. Interestingly, the compound exhibits slow relaxation of magnetization and open hysteresis at zero field and therefore SMM behavior. The significant magnetic exchange coupling found for [{Co(μ-L)}_2_Co] is mainly responsible for the quenching of QTM, which combined with the easy-axis local anisotropy of the Co^II^ ions and the collinearity of their local anisotropy axes with the pseudo-*C*_*3*_ axis favors the observation of SMM behavior at zero field.

## Abstract 2

The simultaneous occurrence of considerable magnetic exchange coupling, strong easy-axis local anisotropy, and collinear arrangement of anisotropic axes in a unique linear Co_3_^II^ complex with triple-phenolate bridging groups leads to the suppression of QTM and the observation of slow magnetic relaxation and open hysteresis under zero field.

## Introduction

During the last three decades, the study of single-molecule magnets (SMMs) has been one of the most active and rapidly developing areas of research in the field of molecular magnetism.^1^ SMMs are open-shell metal coordination compounds that retain their magnetization after eliminating the polarizing magnetic field below the so-called blocking temperature (*T*_B_). In the beginning, the investigation in this area mainly focused on large-spin ground-state metal clusters; however, in recent years, considerable research efforts have been devoted to mononuclear complexes with only one spin carrier, also called mononuclear single-molecule magnets (MSMMs) or single-ion magnets (SIMs).^2^ This is because, in these simple systems, the magnetic anisotropy, which is a key factor for observing SMM behavior, can be deliberately controlled by the design of the ligands field.^3^ Among lanthanide and transition-metal ions, Kramers ions, such as Dy(III) and Co(II), have attracted much attention for constructing coordination compounds with high axial symmetry, large easy-axis magnetic anisotropy (this latter arising from the combined effects of the spin–orbit coupling and the ligands field), and efficient MSMM behavior.^2^ In these compounds, the magnetization of the ground state relaxes through interaction with lattice vibrations (spin–phonon interactions). This process generally requires overcoming an activation energy barrier, *U*_eff_, that largely depends on the magnetic anisotropy (Orbach relaxation process).^1^ The efficacy of the crystal-field-directed strategy to increase axial anisotropy and *U*_eff_ has been demonstrated by the preparation of linear Dy(III)- and Co(II)-based MSMMs with *U*_eff_ and *T*_B_ as high as 1541 cm^–1^ and 80 K, respectively, for the former^4^ and *a U*_eff_ of up to 450 cm^–1^ for the latter.^5^ It should be noted that, in addition to the Orbach relaxation process, other underbarrier processes may contribute to magnetic relaxation, leading to relaxation times faster and *T*_B_ smaller than those expected from the *U*_eff_ values.^1d^ In this regard, it is of crucial importance for observing slow magnetization relaxation and SMM behavior the suppression of the fast quantum tunneling of the magnetization (QTM) occurring within the ground state. This ground-state QTM can be triggered by transverse anisotropy, which is favored by the distortion of the perfect axial symmetry, and intermolecular and hyperfine interactions.^1,2^ In order to suppress the QTM, apart from achieving an almost perfect axial symmetry, intermolecular dipolar interactions could be eliminated by magnetic dilution, and, if possible, metal ion isotopes with zero nuclear spin angular momentum could be used to eliminate potential hyperfine interactions.^6^ Even after accomplishing these conditions, QTM, thermal-activated QTM (TA-QTM), and Raman processes can occur at low temperatures, which limits the magnetization lifetime. To overcome this problem, two additional approaches have been proposed: (i) engineering of molecular vibrations by designing more rigid molecular structures^7^ and (ii) strong magnetic exchange between neighboring magnetic centers.^1g^ With regard to this second approach, it has been observed that, in certain cases, the magnetic coupling between the spin carriers in polynuclear and metal–radical complexes slows down the magnetic relaxation, allowing the observation of SMM behavior.^1g^ Among the systems containing 4f metal ions, this behavior has been mostly observed in 3d–4f polynuclear SMMs with relatively strong ferromagnetic or antiferromagnetic interactions between neighboring 3d and 4f metal ions,^8^ in 4f radical systems, which are characterized by very strong antiferromagnetic interactions,^9^ and 4f polynuclear complexes.^10^ These latter complexes generally present weak magnetic interactions between the 4f ions, which are usually ferromagnetic in nature. However, in some cases, with either carbon-based bridged ligands or metal–metal bonds in mixed-valence dilanthanide complexes, magnetic interactions are significantly enhanced, leading to hard or even ultrahard SMM behavior.^ ^10b,10d^^ It is worth mentioning that, in most cases, 3d/4f and 4f/4f interactions do not suppress the QTM, particularly when the magnetic interactions are weak and, as a result, the exchange-coupled multiplets are close in energy. In addition, it has been observed that magnetic interactions aligning the individual anisotropic axes with the high-order symmetry axis favor the suppression of the QTM and improve SMM properties.^ ^8f,10c^^

**Figure 1:**
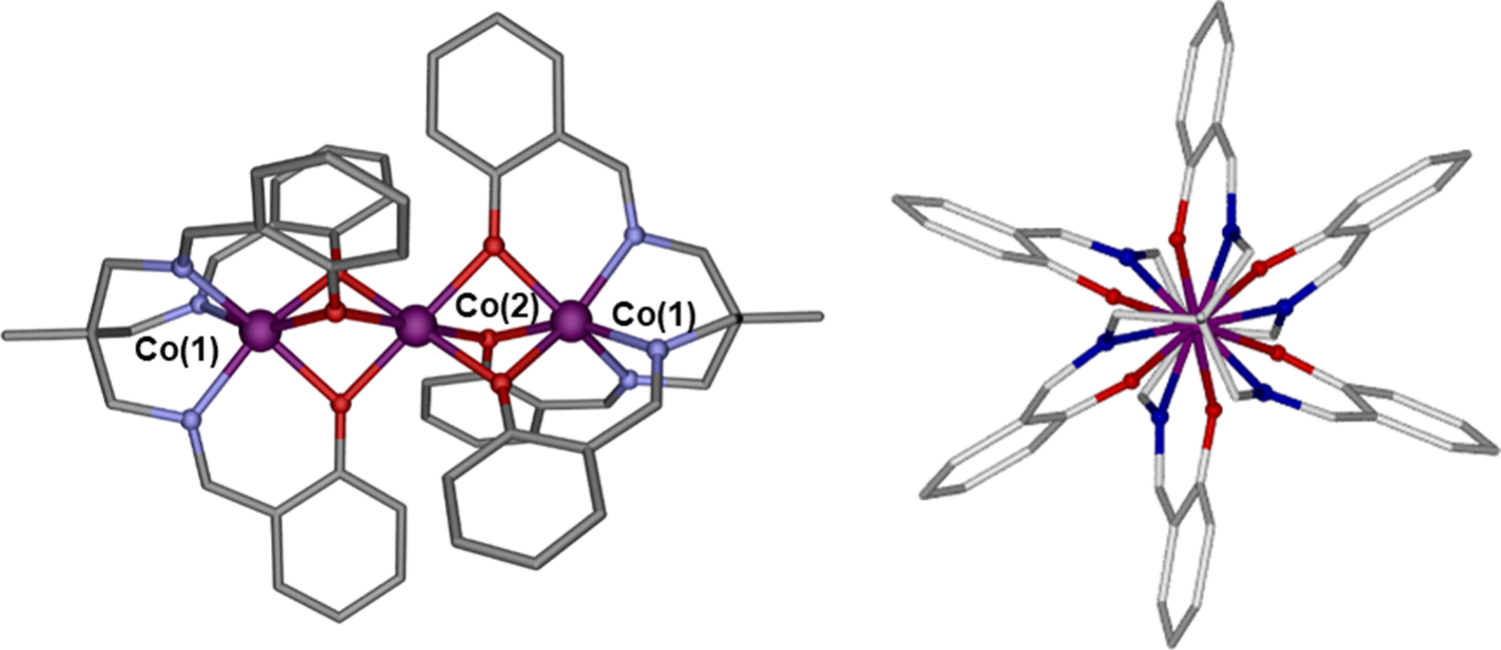
(Left) Molecular structure of **1**. (Right) View along the pseudo*-C*_*3*_ axis showing the paddle-wheel arrangement of the ligands.

**Scheme 1:**
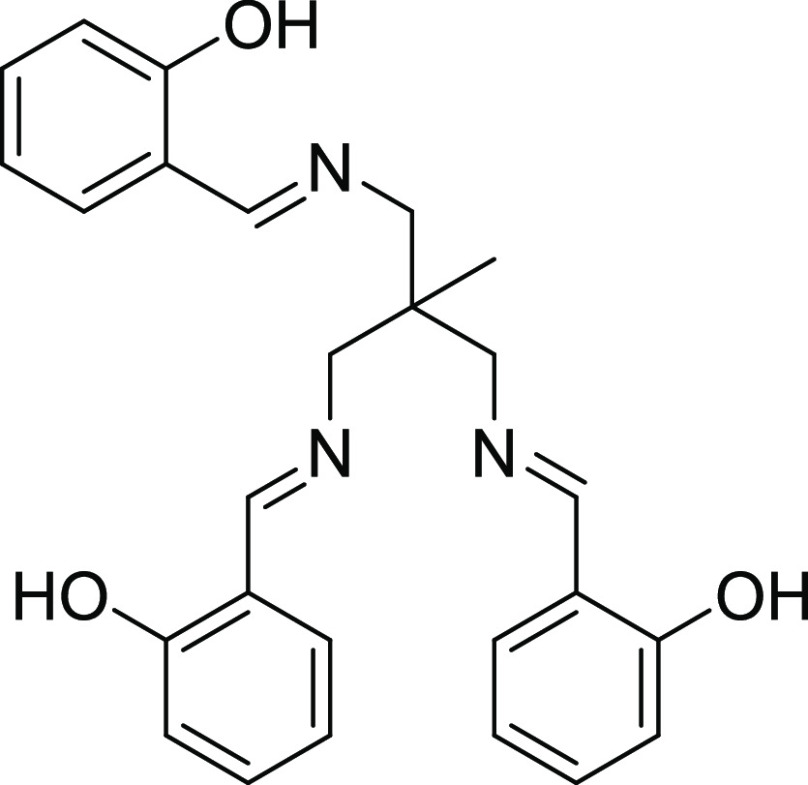
Structure of the H_3_L Ligand

It is worth noting that the examples of QTM suppression in transition-metal clusters, leading to a concomitant activation of the SMM properties at zero magnetic field, are rather scarce and have been observed for compounds exhibiting intermolecular and intramolecular magnetic exchange interactions.^11^ Recently, a very efficient mononuclear tetrahedral Co(II)-based SMM with strong easy-axis anisotropy has been used as a building block to afford an air-stable linear Co(II)–radical–Co(II)-based SMM.^12^ In this compound, the strong magnetic exchange interaction between the spin carriers radically slows magnetization relaxation. Inspired by this strategy, we decided to assemble latent high easy-axis anisotropic trigonal prismatic Co(II) mononuclear building blocks, containing the triply deprotonated tripodal ligand H_3_L (Scheme 1), with Co(II) ions to produce a linear Co_3_ complex [{Co(μ-L)}_2_Co] (**1**) containing triple phenoxide bridging groups between each couple of Co(II) ions (Figure 1). In fact, similar Co(II)–Ln(III)–Co(II) complexes (Ln(III) = Gd and Y) containing two L1^3–^ bridging ligands (H_3_L1 is the same tripodal ligand as the H_3_L ligand but having an additional methoxy group in the ortho position to the phenol group) between the Co(II) and Ln(III) ions and exhibiting similar SMM behavior at zero field have been prepared by following the same strategy.^8f^ It is worth noting that some of us and others^ ^3a,13^^ have recently reported that mononuclear trigonal prismatic Co(II) complexes [Co(L2)]X*_n_* (L2 = tris(pyridylhydrazonyl)phosphorylsulfide tripodal ligand); X = CoCl_4_^2–^, ZnCl_4_^2–^, *n* = 1; BF_4_^–^, ClO_4_^–^, *n* = 2) and [Co(L3)]X_2_ (L3 = tris(1-methylimidazolehydrazonyl)phosphorylsulfide tripodal ligand); X = BF_4_^–^, ClO_4_^–^) exhibit strong easy-axis magnetic anisotropy with an energy gap between the two low-lying Kramers doublets (KDs) arising from the *S* = 3/2 level of about 200 cm^–1^. In view of this, it is expected that the trigonal prismatic mononuclear Co(II) building block generated in situ during the formation of the Co_3_ complex also presents strong easy-axis axial anisotropy. The aim of this work is to know whether the magnetic exchange interactions within this Co_3_ system are strong enough to suppress the zero-field QTM observed in the above-indicated related [Co(L2)]X_2_ complexes, thus promoting the SMM behavior and opening of the hysteresis loop in the absence of a magnetic field. Moreover, if the arrangement of the local anisotropic axes were collinear, the effective uniaxial anisotropy of the coupled Co_3_ system would increase, which could help to improve the SMM properties at zero field.

## Experimental Section

All reagents were obtained from commercial sources and used as received. The H_3_L ligand was prepared following previously reported procedures.^14^

### Physical Measurements

Elemental (C, H, and N) analyses were performed on a Leco CHNS-932 microanalyzer. IR spectra of powdered samples were recorded in the 400–4000 cm^–1^ region on a Nicolet 6700 FTIR spectrophotometer using KBr pellets. *Ac* susceptibility measurements were performed on a PPMS-Model 6000 using an oscillating ac field of 3.5 Oe under different applied static fields. Magnetization measurements at 2 K and different magnetic fields were also performed with the PPMS magnetometer, while the magnetic susceptibility measurements were performed with an MPMS3 Quantum Design SQUID-VSM device. The experimental susceptibilities were corrected for the sample holder and diamagnetism of the constituent atoms by using Pascal’s tables. A pellet of the sample cut into very small pieces was placed in the sample holder to prevent any torqueing of the microcrystals. The X-ray powder diffraction (XRPD) patterns were determined on previously grounded single crystals (Figure S1). For data acquisition, a Philips X’PERT powder diffractometer was used with Cu–Kα radiation (λ = 1.5418 Å) over the range 5 < 2θ < 50° with a step size of 0.026° and an acquisition time of 2.5 s per step at 25 °C. Thermogravimetric analysis was performed using a METTLER-TOLEDO model TGA/DSC1 thermal analyzer in synthetic air (80% N_2_, 20% O_2_) flux of 50 cm^3^ min^–1^ at temperatures ranging from room temperature to 800 °C with a heating rate of 10 °C min^–1^ and a sample size of about 5 mg per run. The electrospray ionization mass spectrometry (ESI-MS) spectra were recorded on an LC/Q-TOF with an ESI Agilent Jet Stream ionization source.

The electrospray ionization mass spectrometry (ESI-MS) spectrum and TG thermogravimetric diagram of **1** are given and discussed in the SI.

### Preparation of **1**

The preparation of **1** was carried out under an inert atmosphere using deoxygenated MeOH and MeCN as follows: A methanolic solution (5 mL) containing Co(acac)_2_ (0.045 mmol, 11.6 mg) was added to another solution of H_3_L (0.03 mmol, 12.9 mg) and Et_3_N (0.09 mmol, 0.013 mL) in 3 mL of MeCN. The light orange solution afforded orange single crystals of **1** in a few hours suitable for X-ray crystal structure determination, which were filtered and subsequently washed with methanol and diethyl ether and air-dried. Yield: 68%. Because these crystals progressively lose solvent molecules, in order to perform magnetic measurements, we decided to fully desolvate crystals over P_2_O_5_ in a vacuum desiccator until a constant weight was achieved (at least 1 day).

Anal. Calcd for C_52_H_48_N_6_O_6_Co_3_: C, 60.65; H, 4.70; N, 8.16; Co, 17.17. Found: C, 60.20; H, 5.01; N, 8.05; Co, 16.90 (from thermogravimetric analysis). ν(C–H aryl), 3023 (w); ν(CH_3_), 2895 (w); ν(C=C), 1628–1440 (s); ν(C–O), 1327 (s); δ(C–H), 754.

### Single-Crystal Structure Determination

Suitable crystals of **1** were mounted on a glass fiber and used for data collection. Data for **1** were collected on an Agilent Technologies SuperNova diffractometer (mirror-monochromated Mo Kα radiation, λ = 0.71073 Å) equipped with an Eos CCD detector.

For **1**, data frames were processed using the CrysAlis Pro software package.^15^ In all cases, the structures were solved by direct methods and refined with full-matrix least squares and SHELXL-2014.^16^ Anisotropic temperature factors were assigned to all atoms except for the hydrogen atoms, which are riding the parent atoms with an isotropic temperature factor arbitrarily chosen as 1.2 times of the respective parent. Attempts to solve disorder problems with crystallization solvent molecules failed in complex **1**. Instead, a new set of F^2^ (*hkl*) values was obtained by the SQUEEZE procedure implemented in PLATON-94.^17^

Final R(F), w*R*(F^2^), goodness-of-fit agreement factors and details on the data collection and analysis can be found in Table S1 in the Supporting Information. Selected bond lengths and angles are given in Table S2 in the Supporting Information. The CCDC reference number for **1** is 2285439.

### Computational Methodology

Quantum-chemical calculations were carried out from the crystallographic structure. The electronic structure and magnetic properties have been computed using state-averaged complete active space self-consistent field calculations (SA-CASSCF (7,5)),^18^ followed by the N-electron valence second-order perturbation theory (NEVPT2) method^19^ with the def2-TZVPP basis set,^20^ including the auxiliary basis sets for correlation and Coulomb fitting for all of the atoms. All calculations were done with the ORCA 5.0.2 quantum chemistry program package.^21^ Spin Hamiltonian parameters (*D*, *E*, and *g*-tensor) were computed using the effective Hamiltonian *S* = 3/2. In this case, spin–orbit effects were included using the quasi-degenerate perturbation theory (QDPT).^22,23^ The employed active space includes seven electrons in five 3d orbitals of Co(II) CAS (7,5). We have included all 10 states for the 2*S* + 1 = 4 (quartet) states arising from the ^4^F and ^4^P terms of Co(II) and all of the 40 states for the respective 2*S* + 1 = 2 (duplet) states arising from the ^2^P, ^2^D (twice), ^2^F, ^2^G, and ^2^H terms of the Co(II) ion. ORCA produces two sets of results, CASSCF and NEVPT2. The splitting of d orbitals due to ligand field has been computed with the ab initio ligand field theory (AILFT)^24^ module implemented in the ORCA program package.

In order to estimate the magnitude and nature of magnetic coupling in **1** with an isotropic Hamiltonian, DFT calculations were performed using the Gaussian16 suite of programs^25^ and following a broken symmetry scheme by means of the B3LYP/TZVP pair of the functional/basis set, which is a standard choice for these cases.^26^ Quadratic convergence at different levels was mandatory since different close low-lying states can arise from calculations. We, then, first took the complete centrosymmetric Co_3_ complex in order to calculate both magnetic pathways, *J* (between neighboring Co(II) ions) and *J*′ (between the external Co(II) ions), at the same time from the corresponding equations derived from the energy differences of the calculated states: (1) the high-spin (+ + +) state bearing a multiplicity of 10 (*S* = 9/2), (2) the + + – quartet (*S* = 3/2), and (3) the + – + quartet (*S* = 3/2). The extracted values of *J* and *J*′ are gathered in Table 1. We also used an alternative method to determine the magnetic coupling constants, consisting of the substitution of one of the cobalt atoms by Zn in order to have just two interacting Co(II) ions, allowing us to calculate both *J* values separately, giving rise to two new Co_2_Zn model complexes: (a) a Co–Zn–Co complex, which allowed us to calculate the *J*′ value, and (b) a Co–Co–Zn complex, which allowed us to get the *J* value. For each case, we got a pair of high-spin (septuplet) and low-spin (singlet) states. Calculations using the same functional and basis set couple were carried out with the ORCA 5.0.2 suite of programs, giving very similar results, which are also presented in Table 1.

**Table 1:** Calculated Exchange Coupling Parameters (cm^–1^) between the Co(II) Ions in **1**

	Co_3_		Co_2_/Zn^a^	
program	*J*	*J*′	*J*	*J*′
Gaussian	–5.38	–0.112	–5.87	+0.154
ORCA	–4.58	–0.106	–4.99	–0.169

a Substitution of Co(II) ions by Zn(II) in the outer (giving *J*) and inner (giving *J*′) positions.

### Pulse-Field Magnetization

Low-temperature magnetization measurements were performed by means of a conventional inductive probe in pulsed magnetic fields. The temperature was reached as low as 0.4 K using a ^3^He cryostat.^27^ Polycrystalline specimens were mounted in a capillary tube made of polyimide. Samples of approximately 20 mg were not fixed within the sample tube, and then, they were aligned along the magnetic field direction. Subsequently, a magnetic field was applied several times until the orientation effect was saturated and the magnetization curves obtained in further shots were found to be identical.

## Results and Discussion

The reaction of the H_3_L ligand with Co(acac)_2_ and Et_3_N in a 2:3:6 molar ratio using a deoxygenated mixture of solvents (MeOH/CH_3_CN) and under an inert atmosphere to avoid the formation of undesired Co(III) species led to the trinuclear Co(II) complex (**1**).

### X-ray Crystal Structure

This complex crystallizes in the monoclinic *C*2/*c* space group (crystallographic data and selected bond distances and angles are shown in Tables S1 and S2, respectively), and its structure consists of well-isolated linear trinuclear Co(II)_3_ molecules with a pseudo-*C*_3_ axis (Figure 1). Within these centrosymmetric molecules, with the center of symmetry being located in the central Co(II) ion (hereafter named Co(2)), two fully deprotonated tripodal ligands (L^3–^) coordinate to the Co(II) external ions (hereafter named Co(1)) through the nitrogen imine atoms and the phenolate oxygen atoms, giving rise to a CoN_3_O_3_ coordination environment. The phenolate oxygen atoms of the two L^3–^ coordinated ligands are additionally linked at opposite sides of the central Co(II) ion, leading to perfect linear Co_3_ molecules, where Co(2) and Co(1) ions are connected by triple phenoxide bridging groups and Co(2) exhibits a CoO_6_ coordination sphere. Continuous shape measurements using SHAPE software^28^ (see Table S3) indicate that the coordination sphere of the Co(1) ions is closer to the ideal TPR-6 polyhedron than to the octahedron OC-6 (*S*_TPR-6_ = 3.984 and *S*_OC-6_ = 5.298), with mean Bailar twist angle, θ, of 9.7° and parallel triangular faces. Nevertheless, the Co(2) coordination sphere is much closer to a perfect octahedral geometry (*S*_OC-6_ = 2.470 and *S*_TPR-6_ = 14.306). For the latter, the mean s/h ratio (defined as the mean donor–donor distance across a triangular face divided by the donor–donor distances between the triangular parallel faces) is 0.89, indicating a significant elongation of the octahedron. Therefore, the CoO_6_ coordination sphere can be better considered as an elongated trigonal antiprism. Co–N and Co–O distances are very similar, are found in the 2.083–2.124 and 2.095–2.103 Å ranges, respectively, and are typical of Co(II) complexes with this kind of donor atoms.

The shortest intramolecular Co(1)···Co(2) and Co(1)···Co(1) distances are 2.909 and 5.818 Å, respectively, whereas the shortest intermolecular distance of 7.671 Å occurs between the Co(1) ions of two neighboring molecules. The screw-type coordination of the ligands around the Co(1) ions induces chirality, leading to a Δ(clockwise) – Λ(anticlockwise) configuration. In order to avoid steric hindrance between the arms of the two coordinated ligands, these turn by about 60° to each other, giving rise to a paddle-wheel arrangement of the ligands when viewing the molecule along the pseudo-*C*_3_ intermetallic axis (Figure 1, right), which is typical of linear trinuclear complexes. Molecules along the *b*-axis display a parallel disposition of the pseudo-*C*_3_ axes, whereas the orientation of the pseudo-*C*_3_ axes alternates in a perpendicular manner along the *c*-axis (see Figure S2).

### Static Magnetic Properties

**Figure 2:**
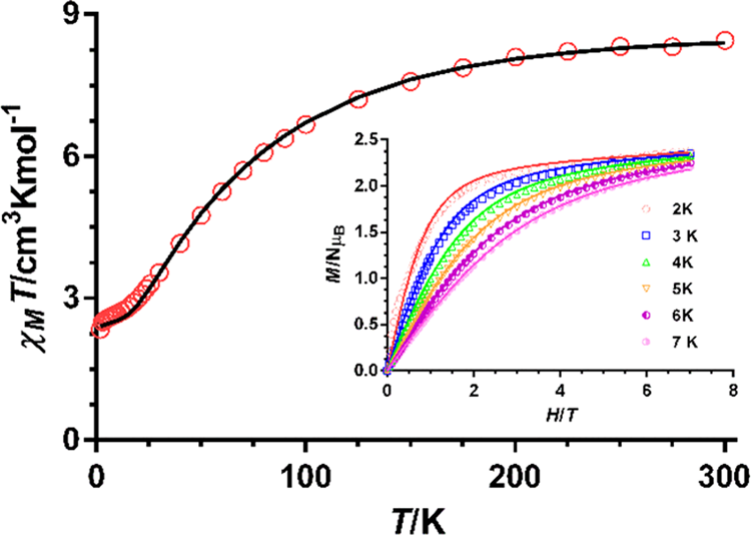
Temperature dependence of χ_M_*T* and field dependence of magnetization (inset) for **1**. The solid lines represent the best fit with the Hamiltonian, given in eq 1.

The temperature dependence of the molar magnetic susceptibility (χ_M_) per trinuclear Co_3_ unit of **1** in the 2–300 K temperature range and under an applied magnetic field of 1000 Oe is given in Figure 2.

The χ_M_*T* value at room temperature of 8.45 cm^3^ mol^–1^ K is much higher than the spin-only value (5.635 cm^3^ mol^–1^ K) for three isolated isotropic Co(II) ions with *g* = 2 and *S* = 3/2, which is indicative of the unquenched orbital contribution of the Co(II) ions. As the temperature is lowered, the χ_M_*T* product decreases first slightly from room temperature to 150 K and then sharply to reach a quasi-plateau of 2.6 cm^3^ mol^–1^ K at 8 K. This decrease is mainly due to substantial antiferromagnetic interactions between the Co(II) ions through the triple phenoxide bridges and the depopulation of the Kramers doublets arising from the spin–orbit coupling (SOC) effects. The field dependence of magnetization up to 7 T in the temperature range of 2–7 K is shown in Figure 2 (inset). The magnetization at 7 T (2.20–2.32 Nμ_B_) is considerably lower than the saturation value expected for a system with *S* = 3/2 and *g* = 2 but is close to the value observed for an isolated highly anisotropic Co(II) ion, resulting from the intratrinuclear antiferromagnetic interactions between Co(II) ions.

The magnetic data were analyzed by the phenomenological approach based on the ZFS of *S* = 3/2 through the following anisotropic spin Hamiltonian.
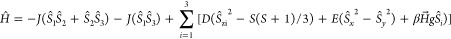
1where the first and second terms account for the intramolecular magnetic couplings, the third and fourth ones correspond to the single-ion axial magnetic anisotropy and the rhombic magnetic anisotropy, respectively, and finally, the fifth term represents the Zeeman interaction. The susceptibility and magnetization data were simultaneously fitted using PHI software^29^ to the above Hamiltonian; however, to avoid overparametrization, *E* and *J*′ were fixed to zero (magnetic measurements have a low sensitivity for determining *E* and |*E*/*D*| parameters and *J*′ is expected to be very small), an axial *g* matrix with *g*_*x*_ = *g*_*y*_ was considered and the same *D*, *g*_*z*_, and *g*_*xy*_ values were assumed for the three Co(II) ions. A very good quality fit was obtained with the following parameters: *J* = −6.38(2) cm^–1^, *g*_*z*_ = 2.938(4), *g*_*xy*_ = 2.167(5), and *D* = −146(2) cm^–1^. It is worth noting that by imposing positive *D* values, the resulting fit was of much worse quality. Moreover, the fit of the magnetic data considering both *J* and *J*′ shows a great correlation between them and tends to have equal values, which is not possible as *J* must be much stronger than *J*′. This was an additional reason for fixing *J*′ = 0.

It is worth noting that Co(II) ions with distorted trigonal prismatic and trigonal antiprismatic coordination spheres, like those observed for Co(1) and Co(2) in **1**, are expected to exhibit significant unquenched orbital angular momentum.^8g^ In view of this, a Hamiltonian that explicitly takes into account this fact, like the Griffith–Figgis (GF) Hamiltonian,^30^ would be, in principle, more appropriate than the SH (eq 1). The GF model uses the T–P isomorphism that considers that the real orbital angular momentum for the ^4^T_1g_ ground state in an ideal Oh geometry is equal to the orbital angular momentum of the ^4^P free ion term multiplied by −3/2; therefore, the ^4^T_1g_ is considered as having an effective orbital moment *L*_eff_ = 1. Although the GF model was developed for octahedral or axially distorted octahedral complexes (square bipyramid), it has been also successfully applied to square-pyramidal distorted complexes.^31^ In this case, the two lowest crystal-field terms derive from the splitting of the ^4^T_1g_; therefore, the T–P isomorphism could be applicable. However, for distorted trigonal prismatic and antiprismatic complexes, where the lowest crystal-field terms derive from the ^4^E ground term,^8g^ this choice is more questionable. Nevertheless, the fact that the Co(1) coordination sphere exhibits an intermediate geometry between trigonal prismatic and octahedron, although a little bit closer to the former, and Co(2) displays a distorted octahedron geometry motivated us to assess the applicability of the GF model in the case of **1**. The Hamiltonian used for analyzing the magnetic data is given in eq 2.
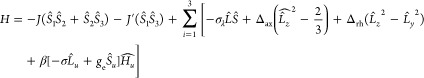
2where *u* = *x*, *y*, *z*; Δ_ax_ and Δ_rh_ represent the splitting of the ^4^T_1g_ (F) ground term due to the axial and rhombic components of the crystal field; λ is the spin–orbit coupling parameter; and *L* and *S* are the orbital and spin angular momentum operators, respectively. This Hamiltonian uses a combined reduction factor, σ = −3/2κ, where −3/2 is a constant required when using T–P isomorphism and κ describes the lowering orbital contribution due to the covalence of the metal–ligand bond and the mixing of the higher energy states into the ground state (as 0 < κ ≤ 1, then 0 > σ ≥ – 3/2). In order to avoid overparametrization when fitting the experimental magnetic data, λ was fixed to the free ion value of 171.5 cm^–1^, *J*′ and Δ_rh_ were fixed to zero (as indicated above, *J*′ should be much weaker than *J*, and for trigonal prismatic and trigonal antiprismatic axial geometries, Δ_rh_ has to be much smaller than Δ_ax_). Moreover, an average σ value was considered for the three Co(II) ions. The axial parameters for the external and central Co(II) ions were named Δ_13_ and Δ_2_, respectively. A good quality fit was obtained with *J* = −7.60 (3) cm^–1^, σ = 1.33(1), Δ_13_=- 2446 (16) cm^–1^, and Δ_2_ = −1241 (8) cm^–1^ (see Figure S3). These values are far away from those extracted with CASSCF/NEVPT2 theoretical calculations (see below) using the GF Hamiltonian (eq 2) of Δ_13_ = −4047 cm^–1^ and Δ_2_ = −1658 cm^–1^. This fact can be due, among other factors, to limitations inherent to the theoretical methods, the unsuitability of the GF model for analyzing Co(II) complexes with trigonal prismatic and antiprismatic geometries, and the simplifications assumed to reduce the number of fitting parameters. Nevertheless, the negative values extracted for parameters Δ_13_ and Δ_2_ point out the strong easy-axis magnetic anisotropy of the Co(II) ions in **1**, which agrees with the results extracted with the spin Hamiltonian (eq 1).

### Theoretical Calculations

Broken-symmetry density functional theory (BS-DFT) calculations were performed to support the *J* value and estimate the magnitude of *J*′. The calculated values are given in Table 1. As can be observed, the calculated *J* values are close to those extracted from the magnetic data, whereas that of *J*′ is very weak and probably antiferromagnetic in nature.

In order to support the easy-axis axial anisotropy of the Co(II) ions fragments in **1**, multiconfigurational ab initio calculations (CASSCF/NEVPT2) based on the experimental X-ray crystal structures were performed using the ORCA 5.0.2 program package^21^ (see Tables S4–S8, SI). The electronic structure of each mononuclear Co(II) fragment of the trinuclear Co_3_ unit was calculated by replacing the other two Co(II) ions with Zn(II) ions. The extracted energies of the spin free states (ligand field terms) for Co(1) and Co(2) ions are given in Table S4. The energy separation values between the ground and first excited states are only 87.8 and 115.4 cm^–1^, respectively, whereas the second excited states for both types of Co(II) ions are above 4000 cm^–1^ and above 1700 cm^–1^ for Co(1) and Co(2), respectively. Therefore, in both cases, the lowest two spin quartets are nearly degenerate so that the Jahn–Teller effect is small and the first spin–orbit coupling (SCO) is operative. As a result, four almost equidistant KDs arise from the SOC, with energy gaps between the ground and first excited KDs at the NEVPT2 level of 260.37 and 243.51 cm^–1^ for Co(1) and Co(2), respectively (Table S5). Since the second excited KD is located at ∼550 cm^–1^ above the ground state, it will be barely populated (∼6%); therefore, the use of an effective zero-field splitting (ZFS) spin Hamiltonian (eq 3) could be appropriate to phenomenologically analyze the theoretical results for each Co(II) fragment.
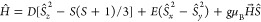
3

The calculated *D* and *E* values using this Hamiltonian are given in Tables 2 and S6, together with the effective *g* values for each doublet projected on a *S* = 1/2 pseudospin. The *D* values are large and negative, as expected for Co(II) ions with trigonal prismatic (Co(1)) and trigonal antiprismatic (Co(2)) geometries and easy-axis axial anisotropy, whereas the effective *g* values confirm the easy-axis anisotropy of the ground state. Nevertheless, the *E*/*D* values and *g*_eff_ values of the ground state indicate a larger rhombicity for Co(2). The anisotropy axes for Co(1) and Co(2) are located along the pseudo-*C*_*3*_ axis passing through the Co(II) ions direction (Figure S4, left), whereas the orientations of the *D*-tensor components are given in Figure S5.

**Table 2:** Computed ZFS Parameter *D*, *E*, |*E*/*D*|, and *g* Values for the Ground State^a^

compound	method	*D* (cm^–1^)	*E*/*D*	*E* (cm^–1^)	δ*E*_1_ (cm^–1^)	Δ*E*_1_ (cm^–1^)	*g*_*x*_, *g*_*y*_, *g*_*z*_^b^
							*g*′_*x*_, *g*′_*y*_, g′_*z*_^c^
Co(1)	CASSCF/NEVPT2	–129.639	0.052933	–6.862	87.8	260.37	1.51, 1.58, 3.34
							0.35, 0.35, 9.35
Co(2)	CASSCF/NEVPT2	–116.222	0.180246	–20.949	115.4	243.51	1.57, 1.80, 3.25
							1.16, 1.24, 8.86

a Co(1) and Co(2) refer to the respective edge and middle Co(II) ions. δ*E*_1_ and Δ*E*_1_ are the calculated first excitation energies before and after considering spin–orbit effects, respectively.

b *g*-Tensor for the true spin *S* = 3/2.

c Effective *g*′-tensors assuming a pseudospin *S* = 1/2.

The largest negative contribution to *D* comes from the first excited quartet state, ^4^Φ_1_ (see Table S7), which is the closest in energy to the ground quartet state. The splitting of the d orbitals for Co(1) and Co(2) (Figure S6 and Table S8) has been calculated by means of the ab initio ligand field method (AILFT)^24^ implemented in ORCA. The first excitation energy involves the transfer of a single electron from the last doubly occupied orbital (d_*xy*_) to the first semioccupied orbital (d_*x*^2^–*y*^2^_) for Co(1) and from the d_*x*^2^–*y*^2^_ orbital to the d_*xy*_ orbital for Co(2), which have the same m_l_ value (±2) and are separated by a small energy of ∼60 cm^–1^. Taking this into account, the *D* value determined qualitatively from the spin allowed part of the second perturbative treatment,^3b^ which depends on the inverse of the excitation energies, is expected to be negative and large (the excitation energy is a little bit larger in Co(2) than in Co(1) and the |*D*| for Co(2) is expected to be slightly smaller). This result agrees well with the sign and magnitude of the theoretically calculated values from the ZFS Hamiltonian.

Interestingly, when the *D* and *E* values for each Co(II) ion are fixed with the values extracted from theoretical calculations, a very good quality fit of the susceptibility and magnetization data was obtained with the following parameters: *J* = −6.26(1) cm^–1^ and *g*_*z*_ = 2.826(2), *g*_*xy*_ = 2.206(4), and *zJ* = −0.014(1) cm^–1^. These parameters are similar to those obtained (see above) by allowing the *D* to vary freely.

It is worth noting at this point that recently Boca et al.^32^ proposed a criterion for quantitatively assessing the suitability of spin Hamiltonian theory (ZFS, eq 1) in octahedral and axially distorted octahedral high-spin Co(II) complexes using theoretical calculations. Based on this criterion, the application of the ZFS model for analyzing the local magnetic anisotropy of the Co(II) ions in **1** is at least problematic. Therefore, the magnitudes of the local extracted values of *D* and *E* should be taken with caution. Even though the use of the SH in the case of trigonal prismatic and antiprismatic Co(II) ions could not be justified because the first-order spin–orbit coupling is operative in both cases,^8g^ it has been extensively applied for analyzing the magnetic anisotropy in trigonal prismatic Co(II) complexes.^13,33^ This is mainly because, as far as we know, there is not any specific model for trigonal prismatic Co(II) complexes, taking into account unquenched orbital momentum. In fact, we have applied the GF model for analyzing the magnetic data of **1** (see above), but the extracted axial splitting parameters for the Co(II) ions (Δ_13_ and Δ_2_) obtained from experimental magnetic data and theoretical calculations are quite different. Therefore, when the GF model is used to analyze the electronic structure of distorted trigonal prismatic and trigonal antiprismatic complexes, the magnitude of the extracted axial splitting parameters should be taken with caution because this model could lead to unreliable results. However, the sign of the magnetic anisotropy using the GF seems to be out of doubt. As a matter of fact, the strong easy-axis magnetic anisotropy found for the Co(II) ions of **1** has been previously observed for other similar trigonal prismatic and trigonal antiprismatic Co(II) complexes, where the sign of the magnetic anisotropy has been supported by EPR or NMR spectroscopy.^13,34^

### Dynamic Magnetic Properties

**Figure 3:**
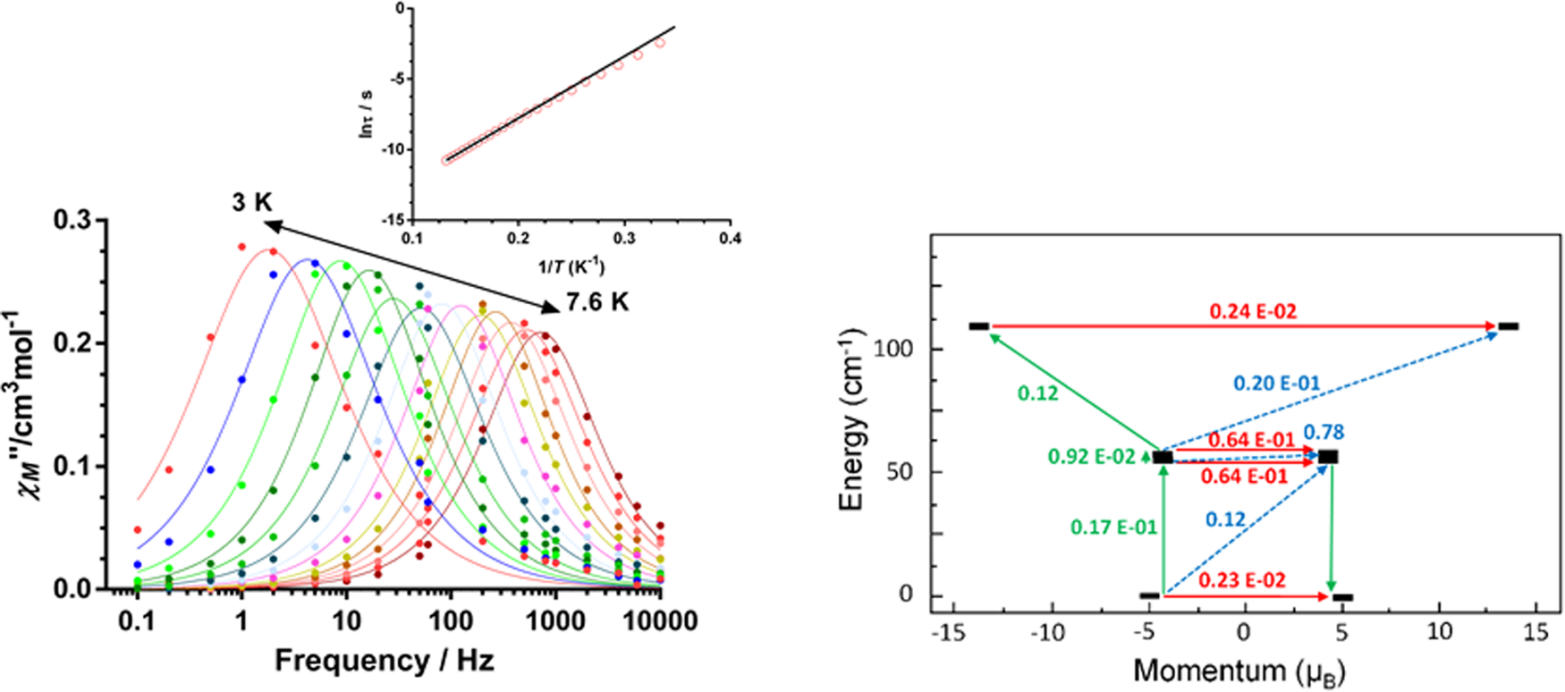
(Left) Frequency dependence of the ac out-of-phase susceptibility (χ_M_″) for **1**. Temperature dependence of the relaxation times in the ln τ vs 1/*T* form (inset). (Right) Ab initio POLY-ANISO-computed magnetization blocking barrier for **1**. The thick black lines represent the four lowest exchange KDs as a function of their magnetic moment along the main anisotropy axis. Green lines indicate the magnetization reversal mechanism. Red lines correspond to QTM and thermally assisted QTM (TA-QTM). Blue dashed lines represent a possible Orbach mechanism. The values close to the arrows indicate the matrix elements of the transition magnetic moments.

Alternating current (ac) measurements at frequencies and temperatures in the 0.1–10,000 Hz and 2–15 K ranges, respectively, were performed to investigate the relaxation dynamics of **1** (Figures 3 and S7–S10). Under zero applied dc field, variable temperature data show frequency-dependent peaks in the 3–7.6 K temperature range without the presence of clearly observable QTM. The α values extracted from the Cole–Cole plots (Figures S9), which are found in the 0.02 (7.6 K)–0.15 (4.4 K), suggest the existence of a unique relaxation process. The high-temperature region of the temperature dependence of the relaxation times (τ), obtained from the fit of the ac data to the generalized Debye model, was represented in the ln τ versus 1/*T* form (Figure 3, inset). As can be observed, the experimental points almost do not deviate from linearity as expected for an Arrhenius law. The fitting of the experimental data in the high-temperature region to equation τ = τ_0_ exp^–*U*_eff_/*k*_B_*T*^, corresponding to an Orbach process, leads to τ_0_ = 6.62 (2) × 10^–8^ s and to thermal energy barrier *U*_eff_ = 43.8(1) K. It should be noted that this *U*_eff_ value is much smaller than the theoretically calculated energy barrier for the local Co(II) ions (∼2*D*).

In view of this, either the magnetic relaxation takes place through a Raman relaxation process or it is not a single ion in origin. The spin relaxation pathways associated with the single-ion Co(II) fragments were calculated for Co(1) and Co(2) using the SINGLE_ANISO code^35^ implemented in the ORCA program package (see Figure S11). The results of this calculation indicate a large tunneling probability in the ground state of Co(1) and Co(2) because the matrix element of the transition magnetic moment within this state of 0.12 μ_B_ and 0.40 μ_B_, respectively, is higher than the required threshold value of 0.1 for an efficient relaxation mechanism.^3b^ These theoretical results do not agree with the experimental ones because **1** shows slow relaxation of the magnetization at zero field. Therefore, it is necessary to go beyond the single ions and consider the exchange interaction between the paramagnetic centers using the POLY_ANISO code^36^ implemented in ORCA. This program employs the Lines model^37^ to fit the experimental susceptibility data using the theoretically calculated energies and wavefunctions of the corresponding ground doublets of the Co(II) fragments. In the present case, due to the strong axiality of the ground KDs of Co(1) and Co(2) sites, the Lines model is fully appropriate. The effective isotropic exchange Hamiltonian is as follows
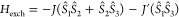
4

The best fitting of the magnetic susceptibility data by fixing *J*′ = 0 (for the same reasons indicated above) led to the magnetic exchange parameters *J* = −13.2 cm^–1^ and *zJ* = −0.2 cm^–1^ (Figure S12). The *zJ* parameter had to be included in the Hamiltonian (eq 4) to take into account the decrease of χ_M_*T* at very low temperatures, essentially due to intermolecular interactions. To connect this *J* value with that obtained from the anisotropic Hamiltonian (eq 1), this latter value must be multiplied by factor 25/9.^38^ In doing so, a *J* value of 17.72 cm^–1^ can be estimated, which is not far from that extracted from the isotropic Hamiltonian (eq 4). It is worth noting that the fitting of the data taking into account *J, J′*, and *zJ* does not significantly change the quality of the fitting and the value of *J* (see Figure S12). When *zJ* or *J*′ and *zJ* are not considered, the *J* value does not change, but the quality of the fit slightly gets worse (Figure S12). The exchange spectrum of **1**, corresponding to the above-fitted exchange parameter, is shown in Figure 3 (right) and consists of eight exchange states grouped into four doublets arising from the Kramers ground state of each Co(II) site (2 × 2 × 2 = 8). The exchange states are arranged according to the values of their magnetic moments, which are the highest in the direction close to the pseudotrigonal axis in **1**. As can be observed in Figure 3 (right), the magnetic moment matrix element for the ground-state exchange doublet is very small; hence, QTM within the ground state is not expected, which is in good accord with the experimental observation of slow relaxation under zero field. However, an Orbach relaxation through the first or second excited states, which are virtually degenerate, could be possible as the matrix element related to the diagonal excitation (0.12 μ_B_) is high enough to allow the spin relaxation through this pathway. The calculated *U*_eff_ value for the relaxation through the first excited state of 55.1 cm^–1^ is not excessively far from the *U*_eff_ value of 30.5 cm^–1^ experimentally extracted from the ac magnetic measurements. This difference between the experimental and theoretically estimated thermal energy barrier can be due to limitations inherent to the theoretical method and possible Raman relaxation through vibrational modes.

**Figure 4:**
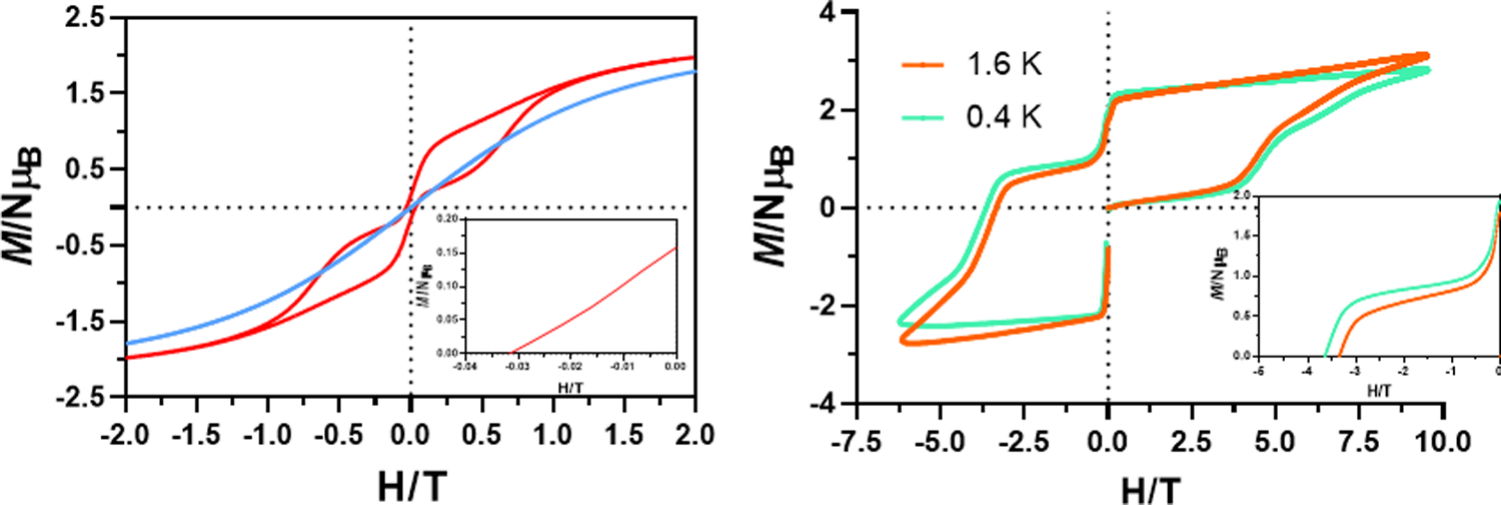
Magnetic hysteresis loops for **1** at 2 K (red) and 3 K (blue) with a 50 Oe/s sweep rate (left). Pulsed-field magnetization curves at a maximum field of 9.5 T and at 0.4 and 1.6 K (right).

Compound **1** was EPR-silent in the frequency range of ca. 100–600 GHz at cryogenic temperatures (5–10 K, Figure S13). The absence of resonances in these conditions is in accord with the energy exchange spectrum of this compound (Figure 3, right) because the intra-Kramers doublet transitions for the ground ΔMs = ± 3/2 KD are forbidden and the first excited state is also a ΔMs = ± 3/2 KD not accessible in energy for the used frequencies. In order to confirm the SMM behavior of **1**, we carried out magnetization hysteresis loop measurements on a powdered sample with a sweep rate of 50 Oe/s in the 2–3 K temperature range. At 2 K, complex **1** shows a pinched at the waist hysteresis loop (Figure 4, left) with small coercive and remnant magnetization values of 320 Oe and 0.2 μ_B_, respectively, which points out the occurrence of effective QTM. The fast QTM relaxation process is mainly triggered by transverse hyperfine interactions between electronic and nuclear (*I* = 7/2) spins. It is worth noting that the presence of open hysteresis above 2 K at zero field in homometallic Co(II)-based SMMs is quite unusual.^5,12,39^ However, at 3 K, no appreciable hysteresis loop was observed at 50 Oe/s. To gain insight into the magnetization dynamics of **1**, we have performed magnetization measurements on a polycrystalline sample using different applied maximum fields in a full-cycle pulsed magnetic field (maximum applied field of 9.5 T) at ^3^He temperature, 0.4 K, and 1.6 K and under adiabatic conditions to minimize the population on thermally activated states (Figure 4, right).^40^ Owing to the extremely fast sweep rates (3.8 T/ms) and lower temperatures used in this kind of measurement, much larger hysteresis loops were observed compared with continuous field measurements. In fact, at 0.4 K, compound **1** exhibits quite large values of the coercive field and remnant magnetization of about 3.6 T and 1 μ_B_, respectively. Moreover, as expected for the SMM behavior, the hysteresis becomes larger when the sweeping rate increases and the temperature decreases.

Several examples of fully magnetostructurally characterized Co(II)_3_ complexes have been reported so far with triangular, bent, and linear geometries.^41^ Most of them contain Co(II) ions with octahedral geometry (for which strong easy-plane local magnetic anisotropy is expected) and weak-to-medium magnetic exchange interactions, both ferromagnetic and antiferromagnetic in nature. Interestingly, only the linear trinuclear Co(II) complexes **1** and [{CoN(SiMe_3_)_2_(μ-η-o-C_6_H_4_(κNSiiPr_3_)_2_])}_2_Co^41d^ have been shown to exhibit slow relaxation of the magnetization at zero field with maxima in out-of-phase ac susceptibility above 2 K. The origin of this behavior could be mainly found in the fact that both complexes contain Co(II) ions with easy-axis magnetic anisotropy. Although the sign and magnitude of the local magnetic anisotropies were not determined for the latter complex, the linear topology and triangular planar coordination geometry of their Co(II) ions point out this type of magnetic anisotropy.^42^ It is worth mentioning that, even though the magnetic exchange coupling is an important factor in suppressing QTM and observing slow relaxation at zero field, it seems to have less influence than the local easy-axis magnetic anisotropy because Co_3_ complexes with stronger magnetic coupling than **1** and [{CoN(SiMe_3_)_2_(μ-η-o-C_6_H_4_(κNSiiPr_3_)_2_])}_2_Co, but without containing easy-axis anisotropic Co(II) ions, do not exhibit zero-field slow magnetic relaxation. Finally, it should be noted that, as far as we know, **1** is the unique example of the Co_3_ complex exhibiting open magnetic hysteresis at zero field. Although the magnetic coupling in **1** is weaker than that in [{CoN(SiMe_3_)_2_(μ-η-o-C_6_H_4_(κNSiiPr_3_)_2_])}_2_Co (*J* = −6.38 cm^–1^ vs *J* = +16.8 cm^–1^ using the “*J*” notation for the Hamiltonian), the former possesses a collinear arrangement of the local anisotropy axes along the pseuso-*C*_*3*_ axis, whereas it seems not to be the case for the latter complex. Therefore, we suggest that the presumable stronger easy-axis magnetic anisotropy of the Co(II) ions in **1**, together with the collinear arrangement of the anisotropy axes, could overcome the effect of the larger magnetic coupling observed in [{CoN(SiMe_3_)_2_(μ-η-o-C_6_H_4_(κNSiiPr_3_)_2_])}_2_Co, thus leading to stronger molecular anisotropy, more effective suppression of the QTM, and the observation of an open hysteresis cycle at zero field.

## Conclusions

A unique linear trinuclear Co_3_ complex has been prepared in situ by self-assembly of the N_6_-tripodal ligand with Co(II) ions. This complex contains strong easy-axis anisotropic Co(II) ions with trigonal prismatic and trigonal antiprismatic geometries and exhibits significant antiferromagnetic exchange interactions between neighboring Co(II) ions through tris(phenolato) bridges. The combination of local easy-axis anisotropies, considerable magnetic exchange coupling, and collinear arrangement of anisotropy axes along the pseudo-*C*_*3*_ axis leads to a more effective QTM suppression and to the observation of slow relaxation of the magnetization and open hysteresis at zero field. More examples of similar Co_3_ compounds with other tripodal ligands are needed to confirm the above hypotheses. Work along this line is in progress in our lab.

## Supporting Information Available

Powder X-ray diffractograms, crystallographic data, bond lengths and angles, magnetic properties (dc and ac), and ab initio theoretical calculations (spin free energy levels, spin–orbit levels, ZFS parameters and *g* tensors, contributions of the excited states to *D*, computed d orbital energy diagrams, blocking barriers) (PDF)
