# Supplementary material for: Zero-Field SMM Behavior Triggered by Magnetic Exchange Interactions and a Collinear Arrangement of Local Anisotropy Axes in a Linear Co3II Complex
Source: Inorg Chem. 2023 Nov 22;62(49):20030–41. doi: 10.1021/acs.inorgchem.3c02817 (PMC10716897; doi:10.1021/acs.inorgchem.3c02817)
Supplement: Supplementary file 1 — ic3c02817_si_001.pdf [file ic3c02817_si_001.pdf]

Supporting Information

Zero-Field SMM behavior triggered by magnetic exchange interactions and collinear arrangement of local anisotropy axes in a linear Co^{II}_3 complex

Andoni Zabala-Lekuona,[a,*] *Aritz Landart-Gereka,*[b] *María Mar Quesada-Moreno,* [†,b] *Antonio J. Mota,*[b] *Ismael F. Díaz-Ortega,* [‡,c] *Hiroyuki Nojiri,*[c] *Jurek Krzystek,*[d] *José M. Seco,*[a,*] *Enrique Colacio*[b,*]

Email Corresponding authors: ecolacio@ugr.es; andoni.zabala@ehu.eus; josemanuel.seco@ehu.eus

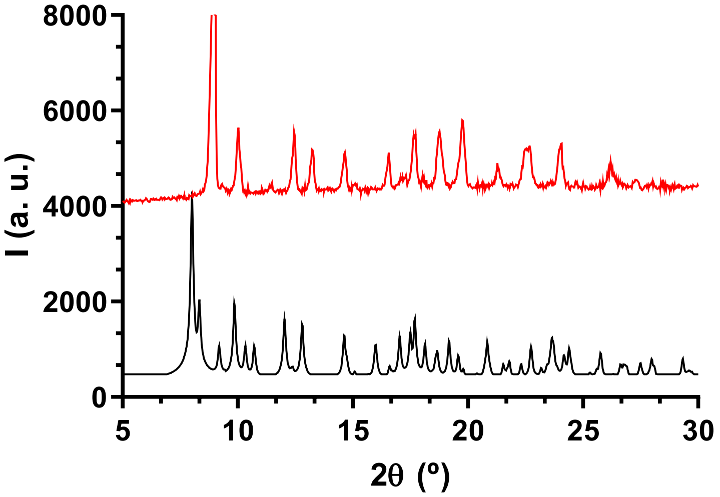

Figure S1.- Simulated pattern from single-crystal X-ray diffraction (black line) and experimental XRPD (red) for compound **1**. The small differences between both diffractograms might be due to the loss of solvent molecules.

Table S1.- Crystallographic data for compound **1**.

**Structure**	**1**
Formula	$C_{52}H_{48}N_6O_6Co_3$
M_r	1029.75
Crystal system	*monoclinic*
Space group (no.)	*C 2/c* (15)
a (Å)	22.2609(7)
b (Å)	10.6725(2)
c (Å)	21.4093(5)
α (°)	90
β (°)	98.166(2)
γ (°)	90
V (Å^3)	5034.8(2)
Z	4
D_c (g cm^{-3})	1.358
μ(MoK$_\alpha$) (mm^{-1})	1.030
T (K)	100.01(10)
Observed reflections	5246 (3649)
R_{int}	0.0820
Parameters	305
GOF	0.950
$R_1^{a,b}$	0.0783 (0.0477)
wR_2^c	0.1092 (0.0965)
Largest difference in peak and hole (e Å^{-3})	0.454 and -0.565

$^aR_1 = \Sigma||F_o| - |F_c||/\Sigma|F_o|$. ^{b}Values in parentheses for reflections with $I > 2\sigma(I)$. $^cwR_2 = \{\Sigma[w(F_o^2 - F_c^2)^2]/\Sigma[w(F_o^2)^2]\}^{½}$

Table S2.- Bond lengths (Å) and angles (°) for compounds **1**.

Symmetry operation: (i) –x+1/2, -y+3/2, -z+1.

**Compound 1**			
Co1-Co2	2.9088(4)		
Co1-O1A	2.072(2)	Co2-O1A	2.1033(19)
Co1-O1B	2.078(2)	Co2-O1B	2.096(2)
Co1-O1C	2.0999(18)	Co2-O1C	2.0948(19)
Co1-N1A	2.124(2)	Co2-O1A(i)	2.1033(19)
Co1-N1B	2.083(3)	Co2-O1B(i)	2.096(2)
Co1-N1C	2.094(3)	Co2-O1C(i)	2.0947(19)
O1A-Co1-O1B	76.92(8)	O1A-Co2-O1B	75.85(8)
O1A-Co1-O1C	78.27(8)	O1A-Co2-O1C	77.69(7)
O1A-Co1-N1A	85.55(8)	O1A-Co2-O1A(i)	180.00(11)
O1A-Co1-N1B	155.94(10)	O1A-Co2-O1B(i)	104.15(8)
O1A-Co1-N1C	113.91(10)	O1A-Co2-O1C(i)	102.31(7)
O1B-Co1-O1C	76.60(8)	O1B-Co2-O1C	76.33(8)
O1B-Co1-N1A	116.68(9)	O1B-Co2-O1A(i)	104.15(8)
O1B-Co1-N1B	86.69(10)	O1B-Co2-O1B(i)	180.0
O1B-Co1-N1C	157.40(9)	O1B-Co2-O1C(i)	103.68(8)
O1C-Co1-N1A	156.24(10)	O1C-Co2-O1A(i)	102.31(7)
O1C-Co1-N1B	115.22(9)	O1C-Co2-O1B(i)	103.67(8)
O1C-Co1-N1C	86.03(9)	O1C-Co2-O1C(i)	180.0
N1A-Co1-N1B	86.27(10)	O1A(i)-Co2-O1B(i)	75.85(8)
N1A-Co1-N1C	84.76(9)	O1A(i)-Co2-O1C(i)	77.69(7)
N1B-Co1-N1C	87.78(11)	O1B(i)-Co2-O1C(i)	76.33(8)
		Co1-O1A-Co2	88.32(8)
		Co1-O1B-Co2	88.34(9)
		Co1-O1C-Co2	87.81(7)

Table S3.- Continuous Shape Measurements for the CoN_3O_3 and CoO_6 coordination environments in compound **1** (the nearer the value to zero, the better fits to an ideal polyhedron).

HP-6 1 D6h Hexagon
PPY-6 2 C5v Pentagonal pyramid
OC-6 3 Oh Octahedron
TPR-6 4 D3h Trigonal prism
JPPY-6 5 C5v Johnson pentagonal pyramid J2

Structure [ML6]	HP-6	PPY-6	OC-6	TPR-6	JPPY-6
**Co(1)**	32.835	19.493	5.298	**3.984**	23.388
**Co(2)**	28.810	28.851	**2.470**	14.306	31.574

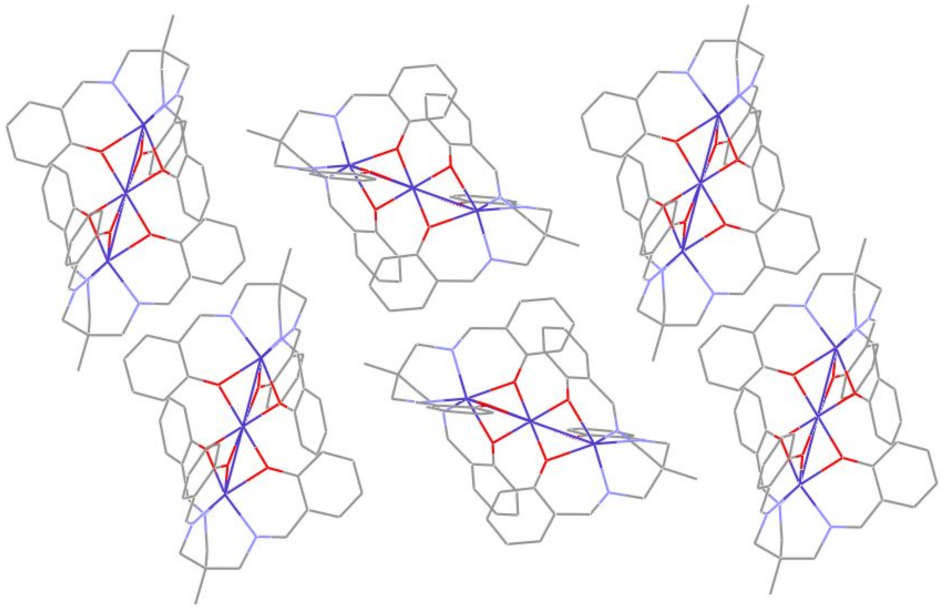

Figure S2.- Orientation of the trinuclear Co_3 molecules of **1** in the crystal.

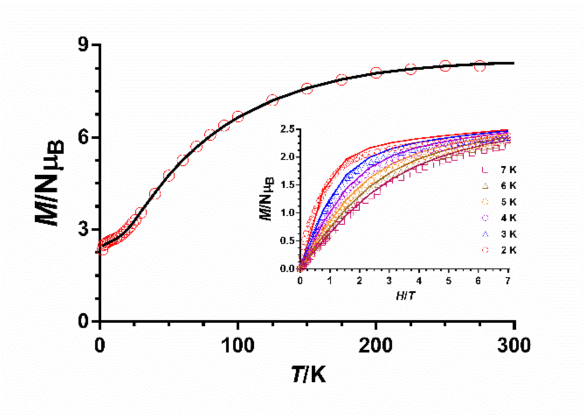

Figure S3.- Temperature dependence of $\chi_M T$ and field dependence of the magnetization (inset) for **1**. The solid lines represent the best fit with the Hamiltonian given in equation 2.

Ab initio Calculations

Calculations were performed for the Co(II) ions in pink in the figure below. The other two metallic ions in blue are Zn(II):

**Co(1)**	**Co(2)**
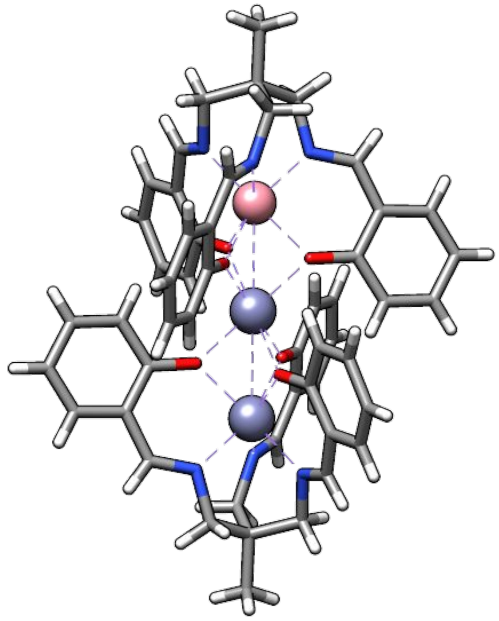	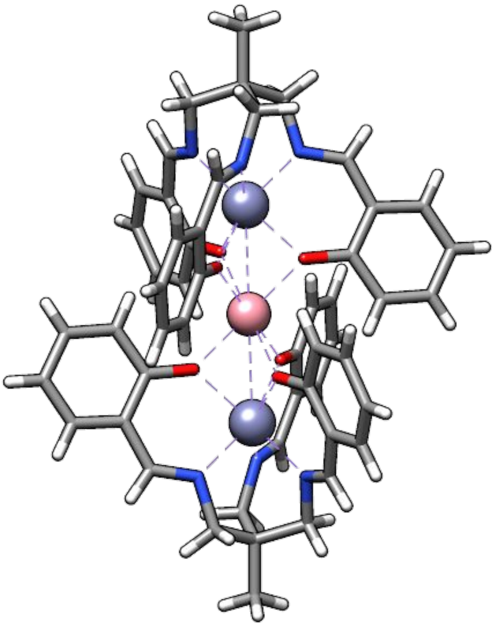

Table S4.- Spin Free CASSCF and CASSCF/NEVPT2 energies (δE, cm^{-1}). **Co(1)** and **Co(2)** refer to the edge and middle Co(II) ions, respectively.

States	**Co(1)**		**Co(2)**	
	**CASSCF**	**NEVPT2**	**CASSCF**	**NEVPT2**
1	0	0	0	0
2	75.0	87.8	88.0	115.4
3	3189.5	4082.8	1407.7	1713.7
4	7023.7	9213.6	6127.3	7796.4
5	7490.2	9862.7	6311.5	8068.7
6	8055.3	10714.9	6410.3	8269.0

Table S5.- Energy levels after the inclusion of spin-orbit effects from CASSCF and CASSCF/NEVPT2 calculations (ΔE, cm^{-1}). **Co(1)** and **Co(2)** refer to the edge and middle Co(II) ions, respectively.

States	**Co(1)**		**Co(2)**	
	**CASSCF**	**NEVPT2**	**CASSCF**	**NEVPT2**
1	0	0	0	0
2	257.27	260.37	243.13	243.51
3	564.77	571.33	533.23	546.22
4	897.96	896.08	886.38	885.10
5	3553.33	4435.56	1825.99	2111.21
6	3639.87	4515.68	1939.16	2213.06
7	7424.69	9596.77	6523.37	8173.27
8	7495.81	9652.28	6594.62	8228.10

[a]Norm of projected states of the effective Hamiltonian from the CASSCF/NEVPT2 calculations are N(KD1) = 0.55 and N(KD2) = 0.64 for Co(1) and N(KD1) = 0.55 and N(KD2) = 0.66.

Table S6.- Computed ZFS parameters D, E, |E/D| and *g* values for the ground state. **Co(1)** and **Co(2)** refer to the respective edge and middle Co(II) ions. **δ**E_1 and ΔE_1 are the calculated first excitation energies before and after considering spin-orbit effects, respectively.

Compound	Method	*D* (cm^{-1})	*E/D*	*E* (cm^{-1})	*δE$_1$* (cm^{-1})	*ΔE$_1$* (cm^{-1})	g_x, g_y, g_z[a] g'_x, g'_y, g'_z[b]
**Co(1)**	CASSCF	-127.744	0.068293	-8.724	75.0	257.27	1.50, 1.59, 3.34<br>0.45, 0.46, 9.34
	CASSCF/ NEVPT2	-129.639	0.052933	-6.862	87.8	260.37	1.51, 1.58, 3.34<br>0.35, 0.35, 9.35
**Co(2)**	CASSCF	-114.155	0.211371	-24.129	88.0	243.13	1.53, 1.79, 3.25<br>1.36, 1.45, 8.75
	CASSCF/ NEVPT2	-116.222	0.180246	-20.949	115.4	243.51	1.57, 1.80, 3.25<br>1.16, 1.24, 8.86

[a] *g*-Tensor for the true spin S = 3/2. [b] Effective *g'*-tensors assuming a pseudospin S = 1/2.

Table S7.- Contributions to *D*-tensor of **Co(1)** and **Co(2)** from CASSCF/NEVPT2 calculations.

	**Co(1)**		**Co(2)**	
	D	*E*	*D*	*E*
$^4\Phi_1$	-148.463	-0.014	-140.359	-0.003
$^4\Phi_2$	6.538	-6.534	15.865	-15.888
$^4\Phi_3$	1.907	-2.279	5.375	-2.424
$^4\Phi_4$	2.989	1.448	3.254	3.885
$^4\Phi_5$	2.862	2.673	-0.178	0.127

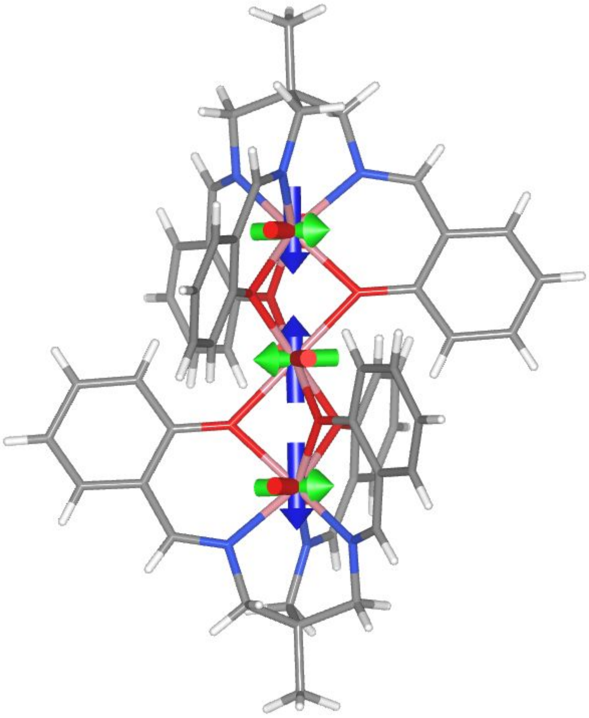

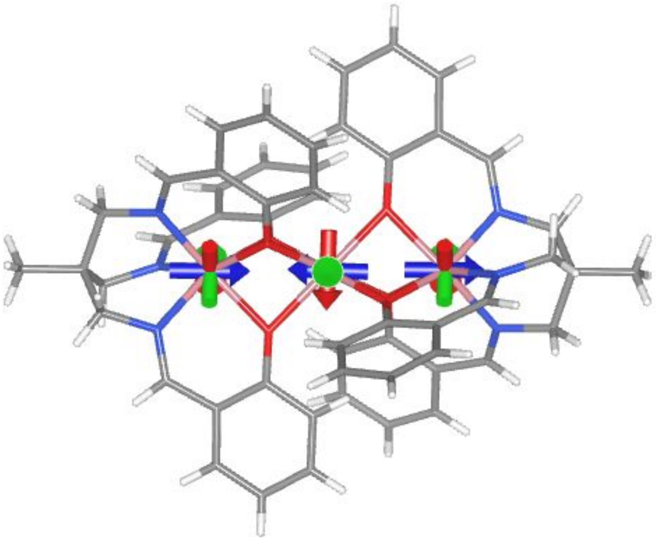

Figure S4.- Orientation of the *g*-tensor components in **Co(1)**, **Co(2) and Co(3)** (same type as **Co(1)**), obtained from CASSCF/NEVPT2 calculations. The reference axis *x*, *y* and *z* of the *g*-tensor are displayed in red, green and blue, respectively.

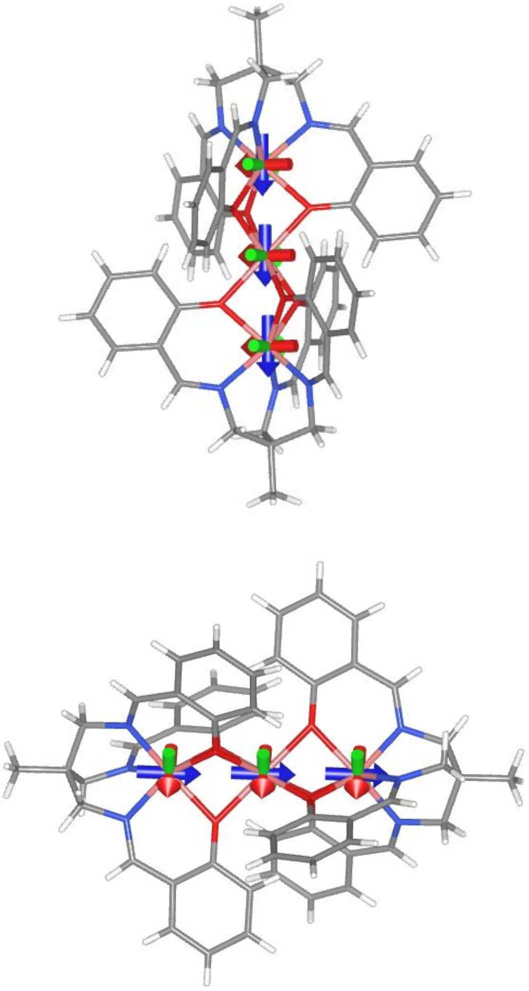

Figure S5.- Orientation of the *D*-tensor components in **Co(1)**, **Co(2) and Co(3)** (same type as **Co(1)**), obtained from CASSCF/NEVPT2 calculations. The reference axis *x*, *y* and *z* of the *D*-tensor are displayed in red, green and blue, respectively.

Table S8.- The ligand field one electron eigenfunctions for **Co(1)** and **Co(2)** from CASSCF/NEVPT2 calculations.

Co(1)

Orbital	Energy (eV)	Energy (cm^{-1})	d_{z^2}	d_{xz}	d_{yz}	$d_{x^2-y^2}$	d_{xy}
1	0.000	0.0	-0.996908	0.054431	-0.025022	0.016394	0.048130
2	0.206	1660.4	0.016964	0.428639	0.139011	0.824459	-0.341942
3	0.213	1716.0	0.063646	0.139221	-0.397531	0.340912	0.838042
4	1.097	8845.8	0.019303	0.362831	-0.829389	-0.203618	-0.372338
5	1.175	9479.3	0.038249	0.813804	0.366241	-0.402886	0.199523

Co(2)

Orbital	Energy (eV)	Energy (cm^{-1})	d_{z^2}	d_{xz}	d_{yz}	$d_{x^2-y^2}$	d_{xy}
1	0.000	0.0	0.948803	-0.064632	0.179990	0.234391	-0.090883
2	0.052	421.3	0.075845	0.453226	0.017652	0.144145	0.876210
3	0.060	486.6	0.300375	-0.067161	-0.478302	-0.808442	0.151370
4	0.897	7237.1	0.014199	0.612296	0.598830	-0.447873	-0.256328
5	0.928	7484.5	-0.059941	-0.641087	0.616378	-0.264792	0.367939

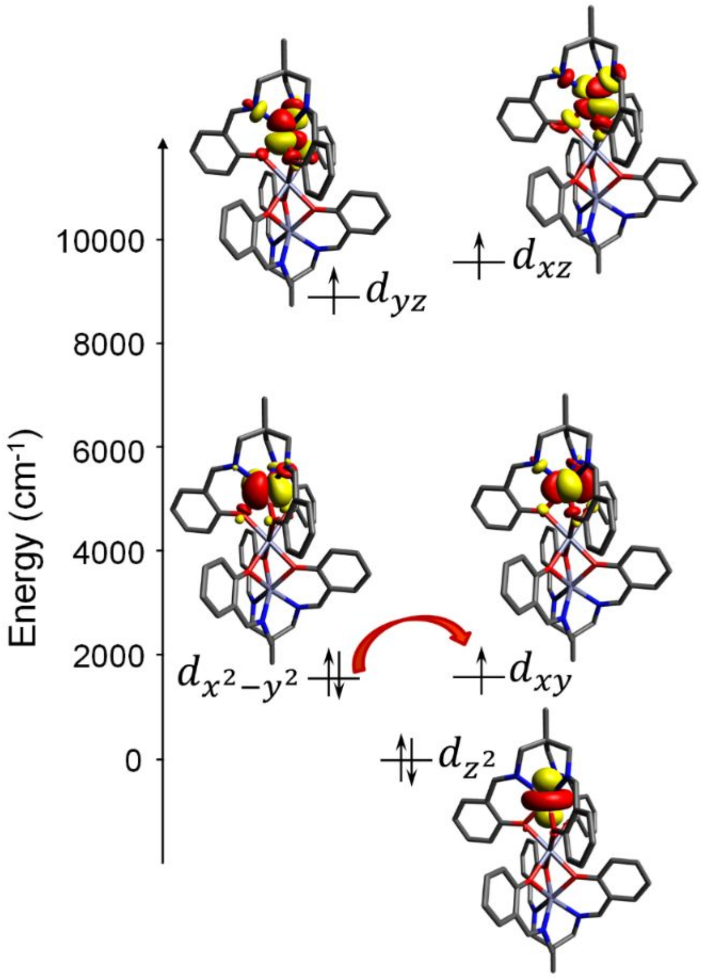

Energy (cm^{-1})
10000
8000
6000
4000
2000
0
d_{yz}
d_{xz}
$d_{x^2-y^2}$
d_{xy}
d_{z^2}

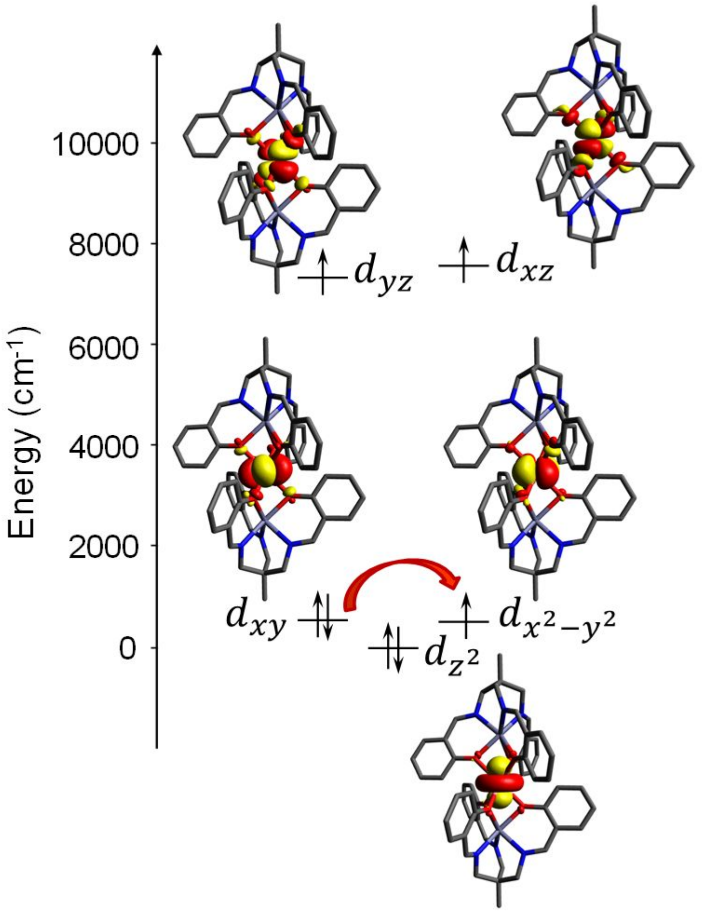

Figure S6.- NEVPT2-AILFT computed d-orbital energy diagram of **Co(1)** (top) and **Co(2)** (bottom). Hydrogen atoms are omitted for the sake of clarity.

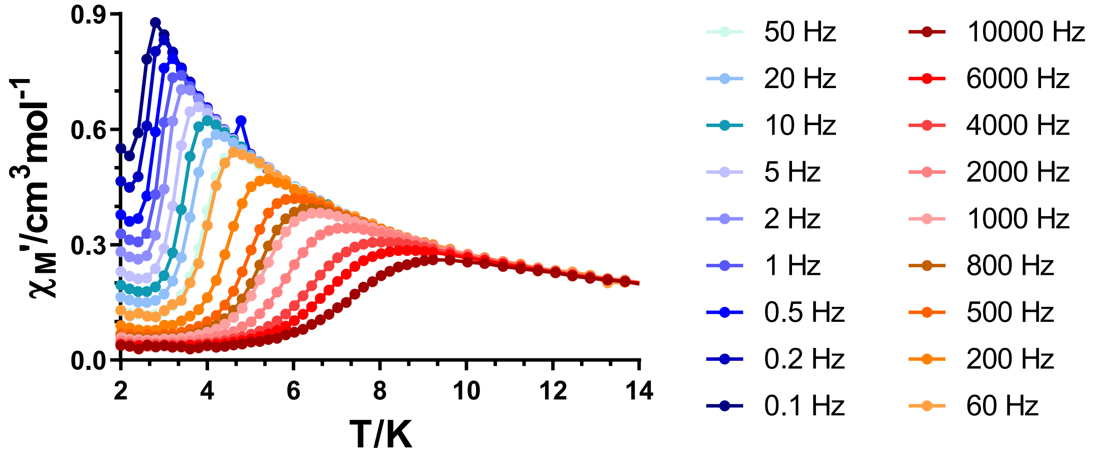

Figure S7.- Temperature dependence of the in phase components of the *ac* susceptibility in a zero applied field for **1**.

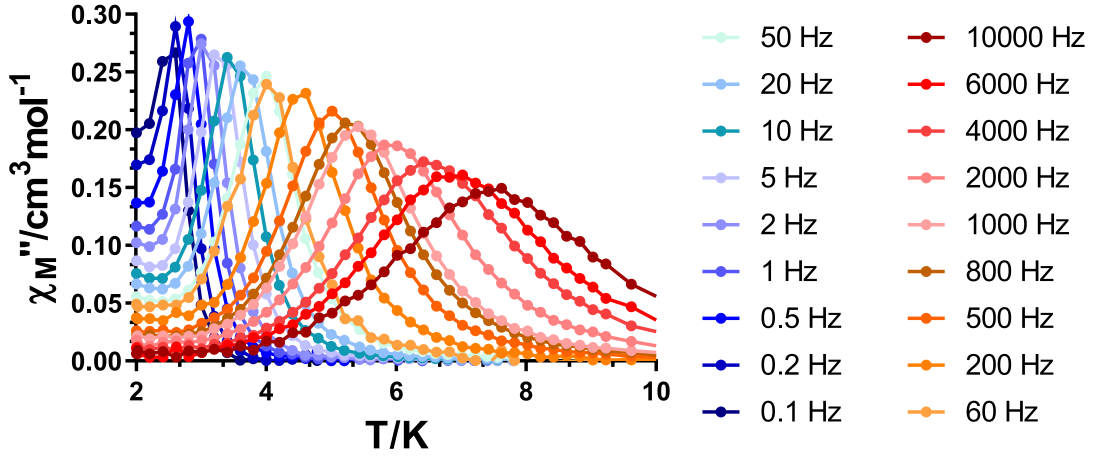

Figure S8.- Temperature dependence of the out-of-phase components of the *ac* susceptibility in a zero applied field for **1**.

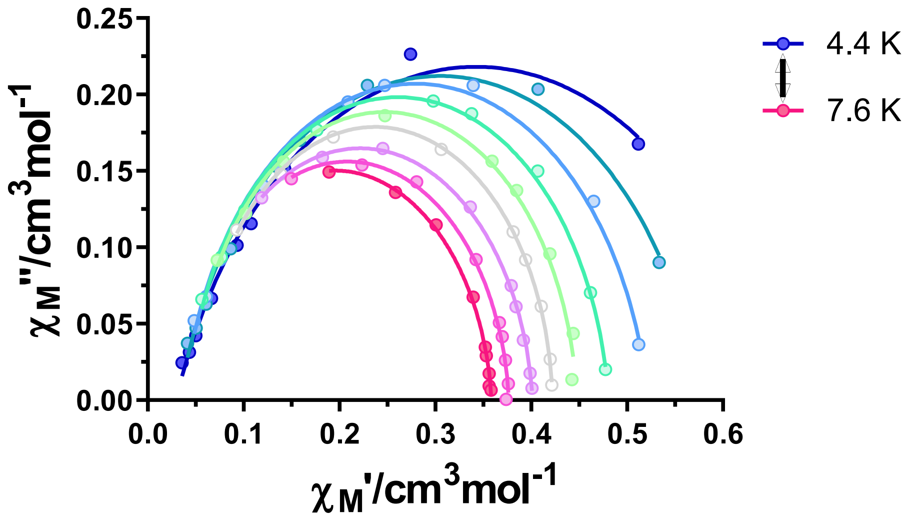

Figure S9.- Cole-Cole plots under zero field for **1**. Solid lines represent the best fit to the generalized Debye model.

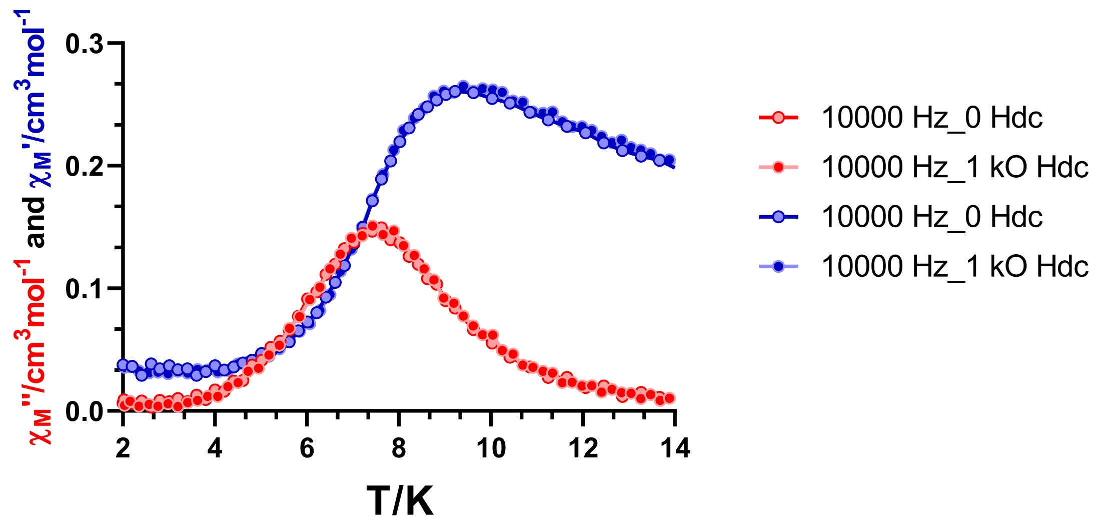

Figure S10.- Temperature dependence of in-phase (blue) and out-of-phase (red) components of the *ac* susceptibility in a zero (light dots) and 1 kOe (dark dots) applied *dc* field for **1**.

Co(1)

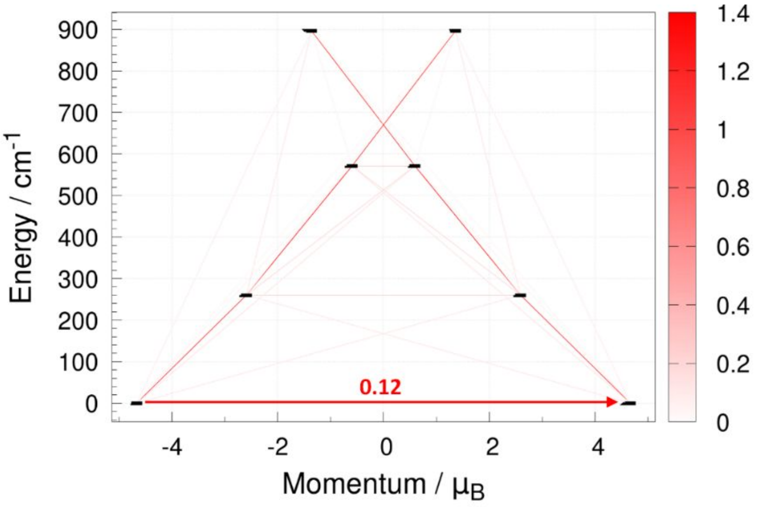

Co(2)

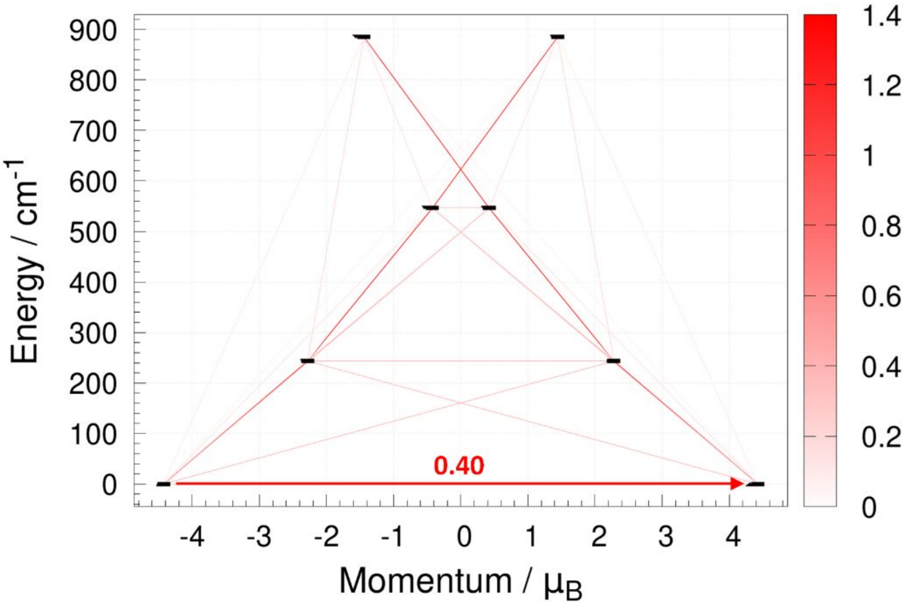

Figure S11.- Possible relaxation pathways in **Co(1)** and **Co(2)** obtained using the SINGLE_ANISO routine. The black lines indicate the KDs as a function of the magnetic moments. Red lines denote QTM in the ground state.

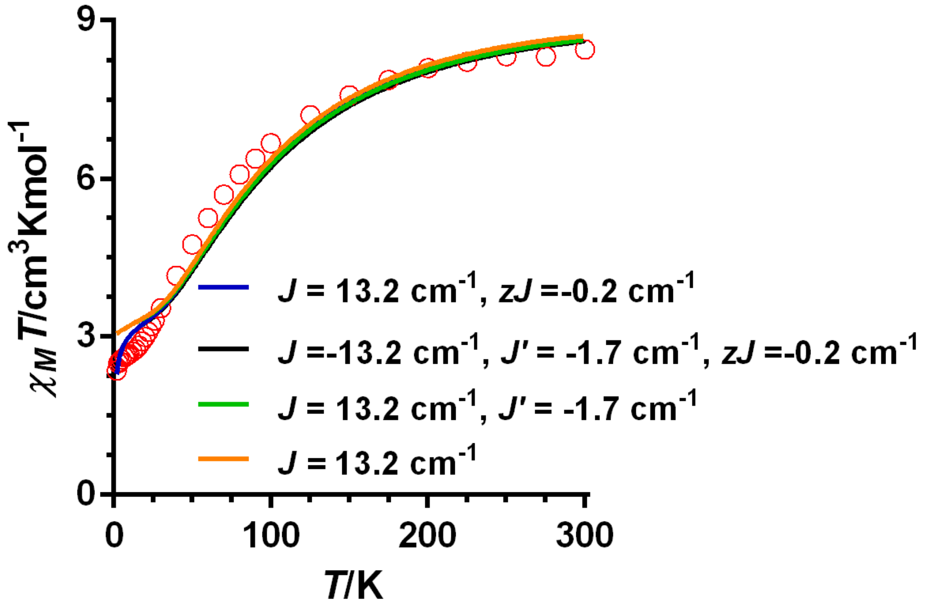

Figure S12.- Temperature dependence of the χ_MT product for **1** (red cicles) and POLY_ANISO fits with the indicated parameters (black solid line).

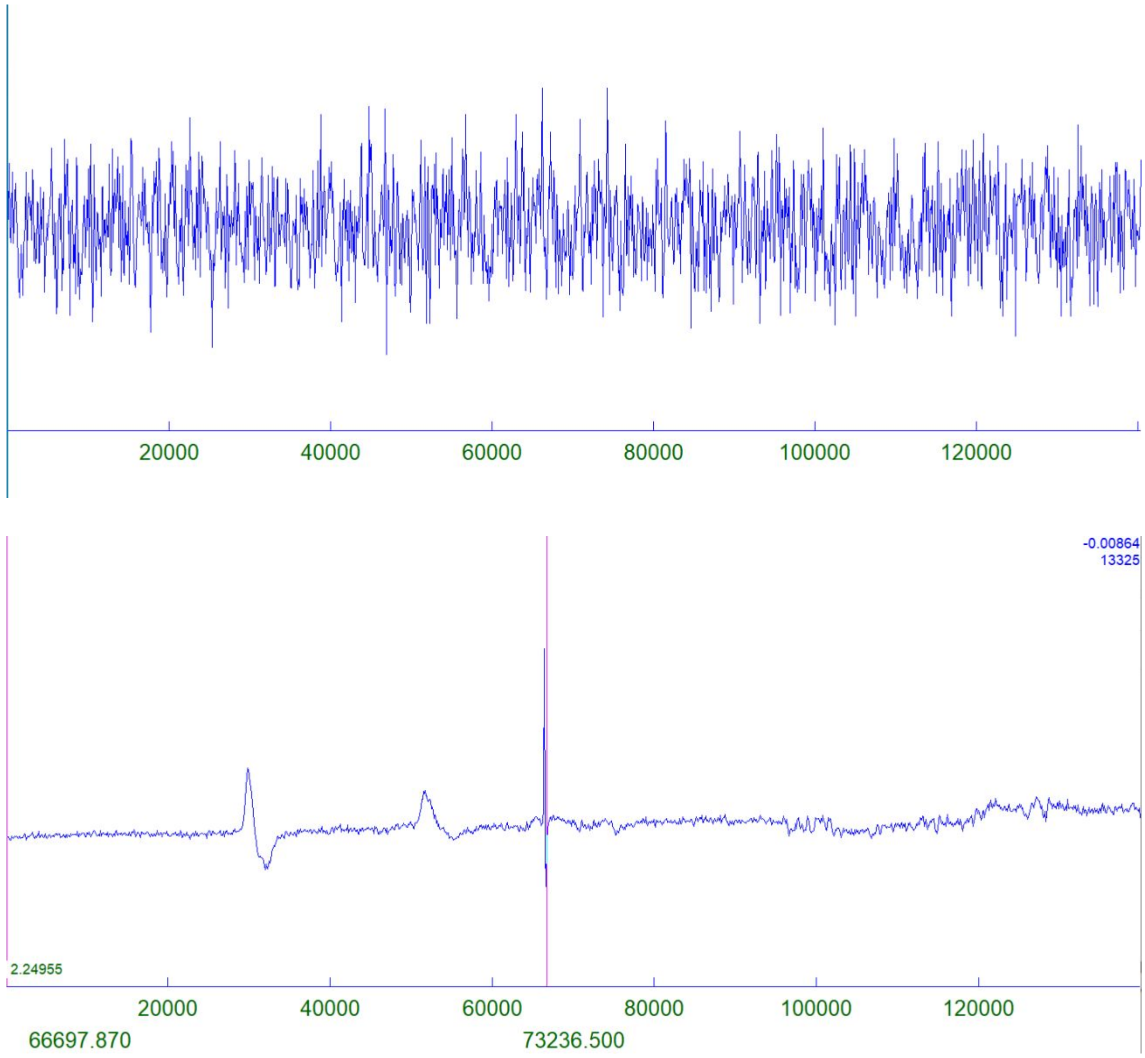

Figure S13.- EPR spectrum of **1** at 4.5 K and 610 GHz (top) and 210 GHz (bottom).

The HF-EPR experiments were run at cryogenic temperatures (5 - 10 K), in the frequency range ca. 100 -- 600 GHz. In the high-frequency regime (in this case 610 GHz), where there is not much power, the spectrum is silent. In the mid-frequency region, where there is most power and hence also the highest sensitivity (in this case 210 GHz,), there is a weak isotropic resonance with g = 2.25, which is most likely just an impurity. The two bumps at ca. 3 and 5 T come from the molecular dioxygen.

The thermogravimetric analysis (Figure S14) confirms that the grinded material contains no solvent as a plateau is observed before starting to decompose. The decomposition process starts below 370 °C and after that CoO is obtained as the residual material. Considering that three equivalents of CoO must be formed and knowing the initial and final mass of the sample, an experimental molecular weight of 977 g/mol was calculated in good agreement, within the error of this technique, to the expected value of 1029 g/mol.

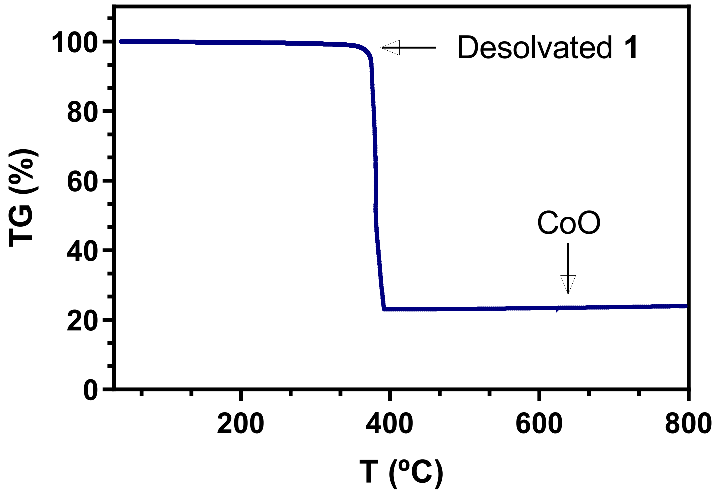

Figure S14.- Thermogravimetric analysis of compound **1**.

A small amount of material was dissolved in methanol and introduced in the instrument, which works with an acid mobile phase (this is an experimental requirement). As it could be observed in Figure S15 (top), the two peaks concerning the monocationic (1029.16) and dicationic (514.58) fragments are properly identified in the spectrum. Additionally, the most abundant peaks with values of 486.12, 430.21, 326.19 and 222.16 (Figure S15, bottom) appear to be due to the presence of the acidic environment. This results in the decomposition and subsequent hydrolysis of the material. Thus, the peak at 486 agrees well with the presence of a mononuclear species of Co(II) with one ligand, the peak at 430 corresponds to the free ligand (H_3L), and, finally, the last two peaks correspond to the singly and doubly hydrolysed fragments of the ligand. The mentioned fragments and the respective mw are shown below the spectra.

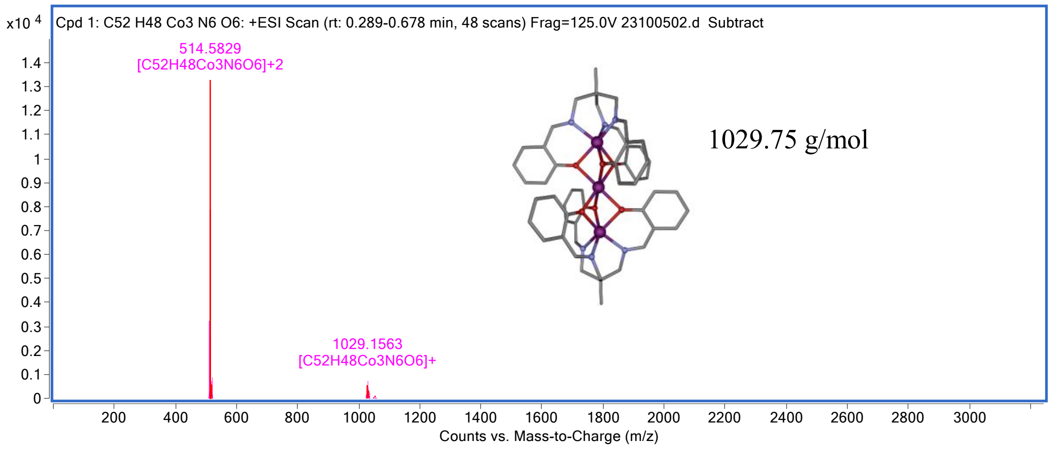

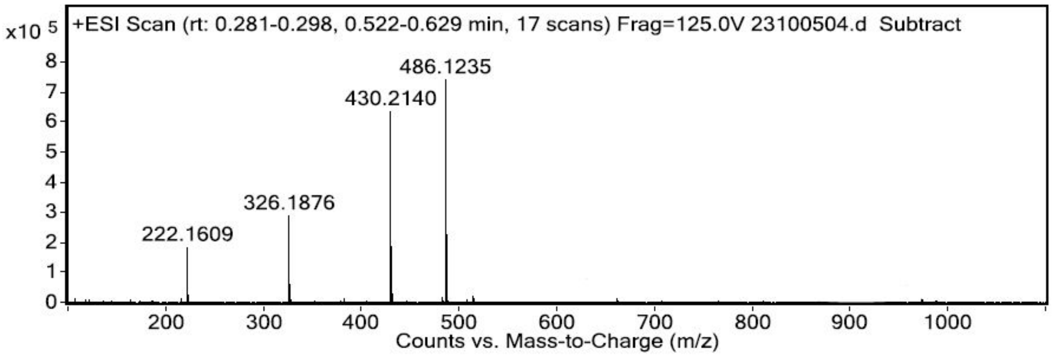

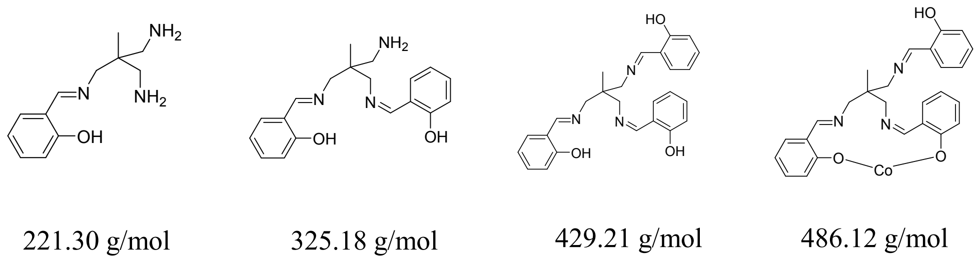

Figure S15.- ESI-MS spectra for **1**. Peaks related to the charged trinuclear compound (top) and peaks related to fragments of **1** due to the acid character of the mobile phase (bottom).
